# Distinct metabolic dependency on BCAA defines cancer stemness and malignancy in human triple-negative breast cancer

**DOI:** 10.1016/j.celrep.2026.117630

**Published:** 2026-07-08

**Authors:** Kenkyo Matsuura, Ririko Shinonaga, Mizuki Yamamoto, Yoshiki Yamamoto, Hsin Chen, Ayaka Maeno, Kayo Okuda, Haruki Inoue, Sota Imotani, John Glushka, Koji Miki, Yukako Watanabe, Mamoru Takada, Hironori Kaji, Jun-ichiro Inoue, Ayuna Hattori, Hiromi Imamura, Takahiro Ito

**Affiliations:** 1 Division of Cell Fate Dynamics and Therapeutics, Institute for Life and Medical Sciences, Kyoto University, Kyoto, Japan; 2 Graduate School of Pharmaceutical Sciences, Kyoto University, Kyoto, Japan; 3 Institute of Medical Sciences, The University of Tokyo, Tokyo, Japan; 4 Graduate School of Medicine, Kyoto University, Kyoto, Japan; 5 Institute for Chemical Research, Kyoto University, Kyoto, Japan; 6 Hacarus Inc., Kyoto, Japan; 7 Complex Carbohydrate Research Center, The University of Georgia, Athens, GA, USA; 8 Department of Energy and Hydrocarbon Chemistry, Graduate School of Engineering, Kyoto University, Kyoto, Japan; 9 Department of Breast Surgery, School of Medicine, International University of Health and Welfare, Chiba, Japan; 10 Department of General Surgery, Graduate School of Medicine, Chiba University, Chiba, Japan; 11 The University of Tokyo Pandemic preparedness, Infection and Advanced Research Center (UTOPIA), Tokyo, Japan; 12 Graduate School of Biostudies, Kyoto University, Kyoto, Japan; 13 Organization for Research Initiatives, Yamaguchi University, Yamaguchi, Japan; 14 Lead contact

## Abstract

Triple-negative breast cancer (TNBC) lacks effective molecularly targeted therapies. Here, we identify branched-chain amino acid (BCAA) metabolism as a selective vulnerability in human TNBC, particularly in the claudin-low subtype. TNBC cells show greater dependence on BCAAs than other breast cancer subtypes, and intracellular BCAA levels are heterogeneous within tumors *in vivo*. Cells with high BCAA levels exhibit enhanced sphere formation and cancer stem cell potential in xenograft models. BCAT1, a cytoplasmic BCAA aminotransferase, is upregulated in claudin-low TNBC and enables tumor growth by promoting BCAA production from branched-chain ketoacids. BCAT1 knockdown impairs TNBC growth *in vivo*, and high BCAT1 expression predicts poor prognosis in patient cohorts. Conversely, BCAA catabolism via the BCKDH complex is suppressed in TNBC, and reactivation of BCKDH by BCKDK knockout blocks clonogenic growth. These findings reveal BCAA metabolic balance as a key regulator of TNBC stemness and malignancy.

## In brief

Triple-negative breast cancer lacks targeted therapies. Matsuura et al. show that claudin-low triple-negative breast cancer cells rely heavily on branched-chain amino acid metabolism to maintain cancer stemness and malignancy. Targeting BCAT1 or reactivating the BCKDH complex disrupts the BCAA-BCKA balance and impairs tumor development, revealing a therapeutic vulnerability.

## Graphical Abstract

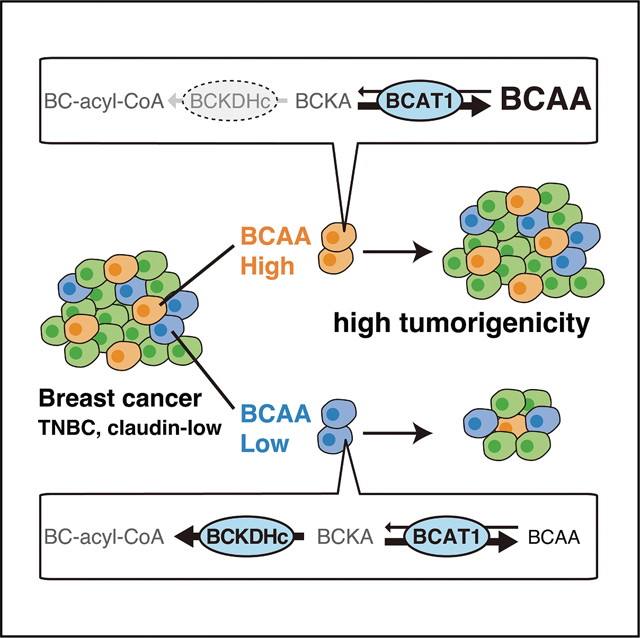

## INTRODUCTION

In malignant transformation and cancer progression, non-genetic events play roles as significant as oncogenic driver mutations do. Altered metabolism is one such mechanism that regulates tumor malignancy. Metabolic reprogramming and rewiring contribute to the disease phenotypes and pathology, including proliferation, differentiation, cell death, therapy resistance, metastasis, and disease progression.^1–4^ Enhanced uptake and usage of glucose, fatty acids, or glutamine contribute to cancer development.^5–7^ Reprogrammed branched-chain amino acid (BCAA) metabolism is an emerging player in driving tumor malignancies. BCAAs, namely valine, leucine, and isoleucine, are alpha-amino acids with a branched-carbohydrate side chain. These amino acids are not only utilized as protein building blocks but also play essential regulatory roles in diverse intracellular events such as autophagy, mRNA translation, stress response, and cell proliferation, which are regulated via BCAA-mediated activation of mTORC1.^8,9^ Considering their multi-faceted roles, it is not surprising that BCAAs are required for cancer development and progression in a variety of human malignancies, including myeloid leukemia, glioma, non-small cell lung carcinoma (NSCLC), and pancreatic ductal adenocarcinoma (PDAC).^10–13^ It remains to be elucidated, however, how BCAA metabolism contributes to tumor aggressiveness and whether intracellular BCAAs directly regulate cancer cell behaviors and functions.

Previous studies have demonstrated the functional roles of BCAAs in cell proliferation, invasion, and tumorigenicity in breast cancer. For instance, among all amino acids, plasma BCAA concentrations are elevated in breast cancer patients when compared with those of healthy subjects. The elevation is also conserved in breast cancer tissues compared with adjacent normal tissues.^14^ In estrogen receptor-positive breast cancer, the intracellular localization of the BCAA transporter LAT1 is regulated by the LLGL2 scaffold protein, contributing to enhanced cell proliferation and tamoxifen resistance.^8^ The BCAA aminotransferase BCAT1 gene is upregulated in and required for antiestrogen-resistant luminal breast cancer cells^15^ and recurrent breast cancer.^16^ Previous studies demonstrated that, in triple-negative breast cancers (TNBCs), BCAT1 regulates PI3K/Akt and Ras/ERK pathways,^17^ and its expression predicts prognosis in TNBC patients.^18^ These studies suggest the contribution of BCAAs and their metabolism in a wide range of breast cancer subtypes. Still, it remains unclear how BCAA regulates the development and pathology of this disease.

In this study, we demonstrate that activated BCAA metabolism is a vulnerability in TNBCs. To investigate how BCAA metabolism regulates TNBC growth and disease properties, we utilized the BCAA biosensor OLIVe,^19^ which enabled us to monitor intracellular BCAA concentrations at the single-cell level. With OLIVe-expressing TNBC cells, we found that intracellular BCAA concentrations are quite heterogeneous within tumor tissue and that TNBC stem cells are enriched in the population with elevated BCAAs *in vivo*. As the underlying mechanism of breast cancer regulation by BCAA metabolism, we revealed that the expression profiles of BCAA metabolic enzymes are distinct among breast cancer subtypes. Notably, BCAT1 is highly expressed in the claudin-low subtype of TNBC (CL-TNBC), and suppression of BCAT1 expression attenuated TNBC cell proliferation. Consistently, CL-TNBC is highly dependent on BCAA for growth. In CL-TNBC cells, BCAT1 catalyzes the BCKA-to-BCAA conversion for anabolic metabolism, supporting tumor growth both *in vitro* and *in vivo*. Finally, we found that the BCKA catabolic enzyme complex, BCKDH complex (BCKDHc), was downregulated in cells with elevated BCAAs. Reactivation of BCKDHc via genetic loss of BCKDK, an inhibitory kinase for BCKDHc, attenuates TNBC proliferation. These results demonstrate the essential role of the BCAA metabolic pathway in driving tumor malignancy and heterogeneity in human TNBC.

## RESULTS

### Subtype-specific dependence on BCAA metabolism in human breast cancers

**Figure 1.**
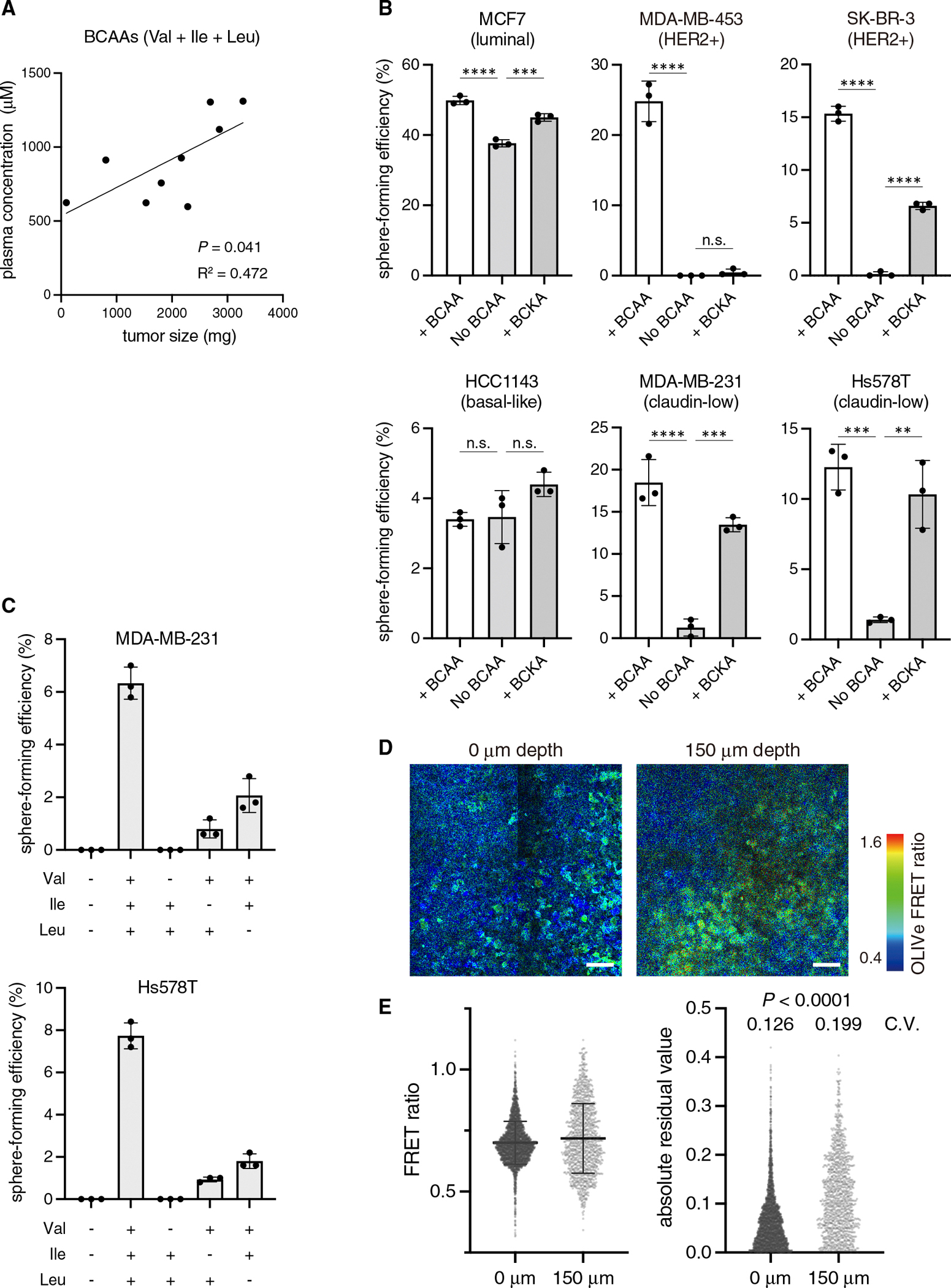
Subtype-specific dependence on BCAA metabolism in human breast cancers (A) The relationship between plasma BCAA concentrations and tumor sizes in xenograft recipients (*n* = 9, black dots). Peripheral blood samples were collected from tumor-bearing mice, and plasma was processed for amino acid measurements. Other amino acid concentrations are shown in Figure S1. Pearson’s correlation coefficient (R^2^) and *p* value are reported. (B) Sphere-forming efficiencies in the presence or absence of BCAAs and BCKAs. Cells were seeded on a low-binding plate in 0.5% methylcellulose growth medium without BCAAs or with either BCAAs or BCKAs. Seven days after seeding, spheres with a diameter of >40 μm were counted as spheres. Sphere numbers were normalized to the number of cells plated and shown as sphere-forming efficiency. *n* = 3 for each condition. Data are presented as mean ± SD. One-way ANOVA with Tukey’s multiple-comparison test was performed. Representative images of spheres are shown in Figure S2. See also Figures S4A and S4B. (C) Sphere-forming efficiencies of MDA-MB-231 and Hs578T cells in medium lacking all or a single BCAA. Sphere-forming efficiency was calculated as described above. *n* = 3 per condition. Data are presented as mean ± SD. See also Figures S4C, S4D, and S4E. (D and E) OLIVe FRET ratio image of a xenograft tumor in a live mouse. (D) A mouse with OLIVe-expressing xenograft tumors formed from MDA-MB-231 cells was anesthetized, and the tumor was imaged using the skin-flap method from the abdominal cavity side. YFP-FRET and CFP signals were collected using two-photon excitation microscopy. The FRET ratio was calculated as the YFP-FRET value divided by the CFP value and presented as a pseudocolor image using an intensity-modulated display mode. The tumor surface area was set to 0 μm for Z-plane optical sectioning. Representative images at depths of 0 and 150 μm are shown. Scale bars represent 100 μm. (E) FRET ratios in individual cells imaged (left). Each dot represents a value from a single cell. The number of cells analyzed was *n* = 3,392 and *n* = 1,186 at depths of 0 and 150 μm, respectively. Absolute residual values were calculated for each cell (right). Data are presented as mean ± SD. Unpaired two-tailed *t* test with Welch’s correction was performed. C.V., coefficient of variation. See also Figure S5. Statistical significance is indicated by asterisks based on *p* values: **p <0.01, ***p <0.005, and ****p <0.001. n.s., not significant.

To assess the contribution of amino acid metabolism in human breast cancer, we first analyzed plasma amino acid levels in an orthotopic xenograft model. We transplanted MDA-MB-231, a human TNBC cell line, into the mammary fat pads of immunodeficient NSG mice and collected blood from tumor-bearing mice for amino acid quantification by fluorescence derivatization and liquid chromatography. Plasma BCAA concentrations were elevated in mice with tumors and positively correlated with tumor size (Figure 1A). Except for proline, other amino acids did not show similar patterns (Figure S1). These findings suggest a functional requirement for BCAAs in TNBC, consistent with previous studies demonstrating elevated plasma BCAAs in human breast cancer and PDAC patients.^14,20^

To determine the functional requirement for BCAA metabolism, we next examined whether BCAAs affect the clonogenic proliferation potential of TNBC cells through sphere formation assays. In the absence of BCAAs, the sphere-forming efficiencies of the claudin-low subtypes MDA-MB-231 and Hs578T were significantly reduced by 14- and 9-fold, respectively (Figures 1B and S2). Similarly, HER2-positive subtypes MDA-MB-453 and SK-BR-3 barely formed spheres in the absence of BCAAs (Figure 1B). In contrast, the luminal type MCF7 and basal-like type HCC1143 breast cancer cells were much less affected by BCAA deprivation (Figure 1B). These results indicate differential BCAA requirements among breast cancer subtypes and a strong dependency on BCAAs for clonogenic growth in the CL-TNBC and HER2-positive cells. Compared with basal-like TNBC, the claudin-low subtypes are mesenchymal and more aggressive, suggesting that metabolic activation and greater BCAA dependence may drive these properties in aggressive TNBCs.^21^

We measured intracellular amino acid levels in breast cancer cells across subtypes (Figure S3). Luminal subtype MCF7 and BT-474 cells, as well as one of the claudin-low subtype Hs578T cells, showed higher intracellular BCAAs than those in HER2-positive MDA-MB-453 and SK-BR-3 cells, basal-like HCC1143 subtype cells, and the claudin-low subtype MDA-MB-231 cells. These findings indicated no clear correlation between cellular BCAA levels and the requirement for BCAAs for sphere-forming efficiency in breast cancer cells (Figures 1B and S3). We also examined whether cell proliferation or survival contributes to the impaired sphere formation in the absence of BCAAs. Consistent with sphere formation assay results, the proliferation of HER2-positive MDA-MB-453 and SK-BR-3 cells, as well as claudin-low MDA-MB-231 cells, decreased under BCAA-depleted conditions (Figure S4A). In contrast, Hs578T proliferation was unaffected by BCAA depletion. Next, we analyzed tumor cell survival in suspension culture conditions using Annexin V staining (Figure S4B). For most cell lines, cell death was not significantly affected by the presence or absence of BCAAs. SK-BR-3 and MDA-MB-231 cells showed higher cell death, which was rescued by the addition of BCKAs.

To determine whether a specific BCAA is necessary for CL-TNBC cells, we performed sphere formation assays in a medium lacking one of the BCAAs. We found that valine depletion completely blocked sphere formation in MDA-MB-231 and Hs578T cells, whereas depletion of either isoleucine or leucine caused less severe yet significant effects on sphere formation (Figure 1C). This result indicates that valine is required for clonogenic growth potential in CL-TNBC cells. To understand the underlying molecular mechanism, we investigated whether previously reported BCAA-related pathways were involved in the phenotype, including mTORC1 activity, epigenetic modification, and reactive oxygen species (ROS) production. Under complete BCAA deprivation, S6K phosphorylation at Thr389 (pT389-S6K) decreased by 60%, compared with the control in complete media (Figure S4C). Removal of valine or leucine resulted in reductions similar to those of BCAA deprivation, whereas isoleucine removal had a minimal effect on pT389-S6K. These findings indicate that valine and leucine contribute more to mTORC1 activation, and the reduced mTORC1 activity partly explains the decreased sphere-forming ability. Histone H3 Lys9 trimethylation (H3K9me3) is a key repressive mark in epigenetic gene regulation. Depletion of all BCAAs increased the modification by 45% (Figure S4D). Removal of valine increased the modification by 30%, and removal of isoleucine or leucine decreased the modification by 2% or 30%, respectively. However, these changes did not clearly correlate with changes in sphere-forming ability, implying no causative effect on CL-TNBC proliferation. Intracellular ROS levels were measured by CellROX dye. In the absence of all BCAAs, about a 2-fold increase in cellular ROS was observed (Figure S4E). In contrast, removal of a single BCAA had little impact on ROS levels. Since the absence of Val completely inhibited sphere formation (Figure 1C), increased ROS generation is unlikely to contribute to the impaired clonogenic growth potential in these TNBC cells.

Next, we took advantage of the BCAA biosensor OLIVe (Figure S5A).^19^ OLIVe consists of seCFP and cp173-Venus (a variant of yellow fluorescent protein [YFP]) connected via a BCAA-binding domain from LIVBP, a bacterial periplasmic BCAA-binding protein.^19^ The BCAA binding to the LIVBP part of OLIVe induces a conformational change, leading to the increased Förster resonance energy transfer (FRET) from seCFP to cp173-Venus, thereby enabling intracellular BCAA quantification via FRET measurements. We generated MDA-MB-231 cells stably expressing OLIVe using the Tol2 transposon system (Figure S5B).^22^ In the presence of BCAAs, the average FRET ratio was around 3.0. The ratio dropped to 2.0 when BCAAs were removed from the culture media and returned to approximately 3.0 when BCAAs were replenished (FiguresS5C and S5D), indicating that FRET signals reflect intracellular BCAA levels in MDA-MB-231 TNBC cells, as shown in HeLa cells.^19^ As in the previous report, when expressed in HeLa cells, the OLIVe FRET ratio is not affected by expression level,^19^ and we observed the same property in MDA-MB-231 TNBC cells (Figure S5E). We measured the OLIVe FRET ratio along with Venus signal intensity, which represents OLIVe protein level. There was no significant correlation between the OLIVe FRET ratio and Venus signal intensity, indicating that the FRET ratio is not influenced by OLIVe protein level. Next, MDA-MB-231/OLIVe cells were transplanted into the mammary fat pads of immunodeficient NSG mice for orthotopic tumor formation. We used two-photon microscopy to detect OLIVe FRET within xenograft tumors. Interestingly, tumor cells with varying FRET ratios were dispersed throughout the tumor tissue, with heterogeneity notably higher in the inner area (150 μm plane) than in the tumor periphery (0 μm; Figures 1D and 1E). Together, our findings suggest that cells with high BCAA levels may be linked to tumor-initiating cell potential *in vivo*.^23^

### Intracellular BCAA levels dictate tumor-initiating potential in CL-TNBC

**Figure 2.**
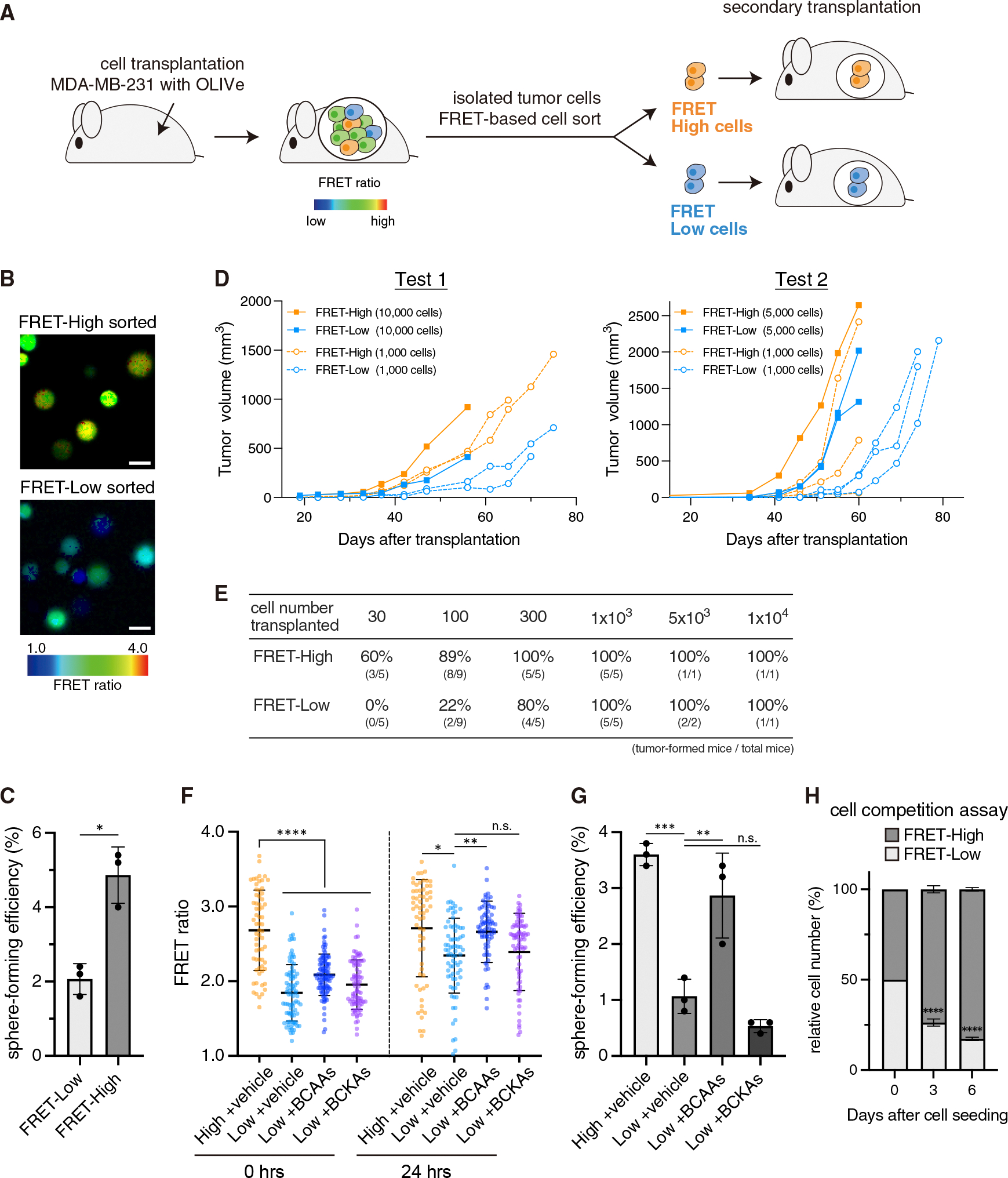
Intracellular BCAA levels dictate tumor-initiating potential in claudin-low TNBC (A) Schematic of BCAA concentration-based cell fractionation and xenograft transplantation. MDA-MB-231 cells expressing OLIVe were transplanted into the mammary fat pad of NSG immunodeficient mice for orthotopic tumor formation. Tumors were dissected, and isolated tumor cells were sorted based on the OLIVe FRET ratio. The top and bottom 10% of cells were sorted as FRET-High and -Low cells, respectively, and then transplanted into new NSG recipient mice. The gating strategy for cell sorting is shown in Figure S6B. See also Figures S6A–S6C. (B) Representative cell images of OLIVe FRET-High and -Low cells after sorting. YFP-FRET and CFP signals were collected using fluorescence microscopy. The FRET ratio was calculated and displayed as a pseudocolor with an intensity-modulated display color, with a range of 1.0–4.0. Scale bars represent 20 μm. See also Figures S6A, S6B, and S6C. (C) Sphere-forming efficiency of FRET-High and -Low cells. 500 cells were seeded in a 0.5% methylcellulose growth medium and cultured for 7 days (*n* = 3 each). Spheres with a diameter greater than 40 μm were counted as spheres, and sphere-forming efficiency was calculated. Data are presented as mean ± SD. Unpaired two-tailed *t* test with Welch’s correction was performed. See also Figures S6F and S6G. (D) Tumor formation by OLIVe FRET-High and -Low cells. Sorted FRET-High and -Low populations were orthotopically transplanted into NSG mice at doses of 1,000 or 10,000 cells (test 1) or 1,000 or 5,000 cells (test 2) per recipient. Tumor volume was measured at intervals of 3 or 4 days after transplantation. Broken lines with empty symbols indicate tumors developed from 1,000 cells (tests 1 and 2); solid lines and filled symbols indicate those from 10,000 cells (test 1) or 5,000 cells (test 2). Each line represents tumor volume in the recipient. (E) *In vivo* limiting dilution assay to assess the tumor-initiating potential of OLIVe FRET-High and -Low cells. Isolated tumor cells were transplanted as described in (D) at the cell numbers shown. Recipients were monitored every 3–4 days for tumor formation. The probability of tumor formation was calculated and shown as a percentage. The number of mice with tumor development and the total number of mice are shown in brackets. (F) Changes in OLIVe FRET ratio with excess BCAAs or BCKAs. Sorted FRET-Low cells were seeded with the indicated metabolites at five times the concentration of standard RPMI 1640 medium. YFP-FRET and CFP signals were collected by fluorescence microscopy as in (B) at 0 and 24 h of imaging. FRET ratios were calculated for each individual cell. At least 60 cell images from each group were analyzed to calculate the OLIVe FRET ratio. Data are presented as mean ± SD. Welch’s ANOVA with Dunnett’s T3 multiple-comparison test was performed. See also Figures S6D and S6E. (G) Sphere-forming efficiency of OLIVe FRET-High and -Low cells with excess BCAAs or BCKAs. Cell conditions were as described in (F), and the sphere formation assay was performed. *n* = 3 per condition. Data are presented as mean ± SD. One-way ANOVA with Tukey’s multiple-comparison test was performed. (H) Cell competition assay between OLIVe FRET-High and -Low cells. Isolated tumor cells were sorted based on OLIVe FRET ratio. FRET-High and -Low cells were labeled with CellTrace Far Red and CellTrace Yellow dyes, respectively. Equal numbers of cells were mixed and co-cultured. On days 3 and 6 of culture, cell populations were analyzed by flow cytometry. *n* = 3 per condition. Data are presented as mean ± SD. One-way ANOVA with Dunnett’s multiple-comparison test was performed. Statistical significance is indicated by asterisks based on *p* values: *p <0.05, **p <0.01, ***p <0.005, and ****p <0.001. n.s., not significant.

Because we found that CL-TNBC cells are highly dependent on BCAAs, we next examined whether BCAAs directly contribute to cancer development *in vivo*. To achieve this goal, we fractionated tumor cell populations based on their FRET ratios, and thereby their intracellular BCAA levels, via cell sorting.^19,24^ The unfractionated cells from the mammary tumors showed a broader distribution of OLIVe FRET signals compared with cells cultured *in vitro* (Figures S6A and S6B), indicating higher heterogeneity in BCAA metabolism *in vivo* than in cells cultured *in vitro*. We next investigated their functional differences by sorting tumor cells into two groups, the top and bottom 10% of cells based on the OLIVe FRET ratios: FRET-High and FRET-Low cells, respectively (Figures 2A, 2B, and S6B). We confirmed that FRET-High cells had significantly higher BCAAs than FRET-Low cells by direct measurement of intracellular amino acids (Figure S6C). When tested for sphere formation *in vitro*, OLIVe FRET-High cells formed 2.5-fold more spheres than OLIVe FRET-Low cells (Figure 2C). We next tested tumor-initiating potential *in vivo*. Upon serial transplantation into NSG mice, OLIVe FRET-High cells formed larger tumors more rapidly than OLIVe FRET-Low cells did (Figure 2D). Moreover, FRET-High cells exhibited greater tumor-initiating cell activity than an equal number of OLIVe FRET-Low cells when transplanted. To assess the frequency of tumor-initiating cells in each population, we conducted an *in vivo* limiting dilution assay (Figure 2E). When 300 or more cells were transplanted, all mice developed tumors with FRET-High cells, whereas not all recipients that received 300 FRET-Low cells developed tumors. With 100 cells transplanted, 89% of recipients developed tumors from FRET-High cells, while only 22% of the mice developed tumors from FRET-Low cells. Only 30 FRET-High cells could still induce tumors in 60% of recipients, whereas FRET-Low cells did not in any recipients tested. These results indicated that intracellular BCAA levels dictate tumor-initiating potential in CL-TNBC.

To investigate whether cellular BCAA levels are stable or dynamic, we conducted time-lapse imaging of FRET-High and -Low cells sorted from xenograft tumors. We analyzed FRET ratios in each cell and tracked their changes over the course of 120 h under standard culture conditions in MDA-MB-231 cells (Figure S6D). Initially, FRET-High cells showed a mean FRET ratio of 2.8; at the end of imaging (120 h), the mean ratio was 2.9, indicating that their BCAA levels remained stable during this period. In contrast, FRET-Low cells started with a mean ratio of 1.9 at time = 0 h, which increased to 2.6 over the 120 h imaging period. Notably, the ratio difference between FRET-High and -Low cells remained statistically significant even at the end of the 120 h time-lapse tracking. These results suggest that (1) differences in BCAA levels are maintained between FRET-High and -Low cells and that (2) BCAA-High cells can sustain high intracellular BCAA levels, whereas FRET-Low cells are more dynamic and susceptible to extracellular environments.

We also examined whether different culture conditions influence intracellular BCAA stability by using human plasma-like medium (HPLM), which contains nutrients at concentrations similar to human plasma.^25^ We observed that the mean ratios of FRET-High and -Low cells behaved similarly in HPLM as in the standard RPMI 1640-based culture medium, suggesting that the culture medium had little, if any, impact on the properties of BCAA-High and -Low cells (Figure S6E). Interestingly, heterogeneity in FRET ratios was greater in HPLM compared with the standard culture condition in both populations, which appears to mirror the greater BCAA heterogeneity observed in xenograft tumors compared to *in vitro*-cultured cells (Figures 1D, 1E, and S6B). Then, we examined whether extracellular BCAAs influence cellular BCAA levels. Sorted FRET-Low cells were cultured with excess BCAAs (five times the concentrations in standard RPMI 1640 medium) for imaging (Figure 2F). After 24 h with excess BCAAs, the FRET ratio went up to a level comparable to that of the FRET-High cells. Consistent with the increase in the FRET ratio, BCAA supplementation enhanced the sphere-forming ability of FRET-Low cells (Figure 2G). Furthermore, when sorted FRET-High and -Low cells were labeled with different cell tracker dyes and co-cultured, FRET-High cells became more dominant over a 6-day culture (Figure 2H), suggesting that CL-TNBC cells with higher BCAAs have a competitive growth advantage over those with lower intracellular BCAAs. When we tested cell proliferation, no significant differences were observed between FRET-High and -Low cells (Figure S6F). In contrast, cell death was significantly lower in FRET-High cells compared with FRET-Low cells (Figure S6G). These results suggest that suppression of cell death may contribute to the enhanced clonogenic growth and tumor-initiating potential of FRET-High cells.

### The BCAA aminotransferase BCAT1 is indispensable for CL-TNBC

**Figure 3.**
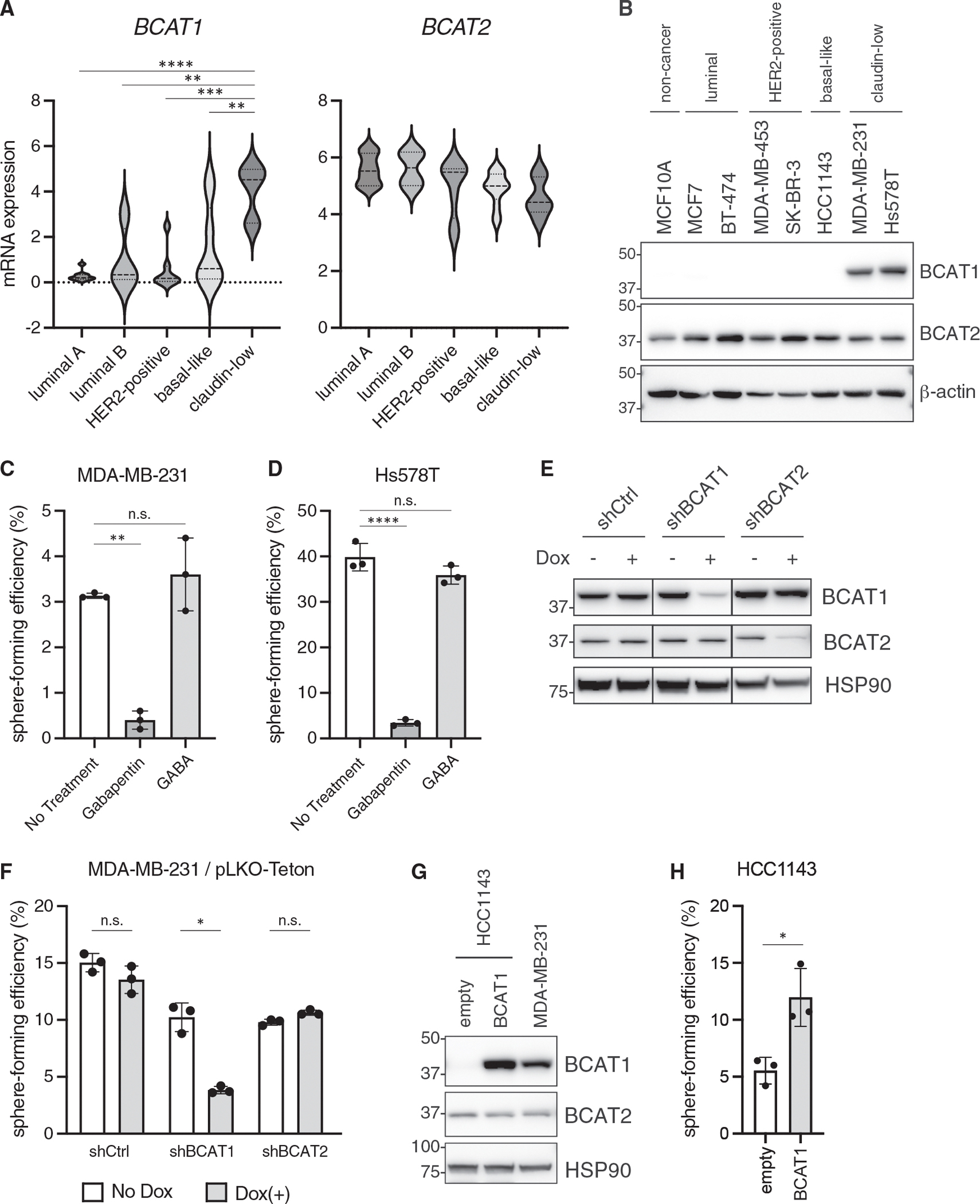
The BCAA aminotransferase BCAT1 is indispensable for claudin-low TNBC (A) Expression of BCAT genes in breast cancer cell lines. The mRNA expression scores for human BCAT1 and BCAT2 were analyzed across the luminal A, luminal B, HER2-positive, basal-like, and claudin-low subtypes using the Cancer Cell Line Encyclopedia (CCLE) dataset. Subtype classification details are provided in the STAR Methods section. Data are shown as violin plots with the median (dashed line) and interquartile range (dotted lines). One-way ANOVA with Tukey’s multiple-comparison test was performed. (B) BCAT protein expression in breast cancer cells. Cell lysates from each cell line were prepared and subjected to immunoblot analysis for BCAT1 and BCAT2 proteins. β-Actin served as a loading control. The numbers on the left indicate molecular weight in kDa. See also Figures S8A, S8B, and S8C. (C and D) Sphere-forming efficiency of claudin-low cell lines after treatment with a BCAT1 inhibitor. Claudin-low subtype breast cancer cells, either MDA-MB-231 (C) or Hs578T (D), were cultured in 0.5% methylcellulose medium, with or without 20 mM gabapentin (the BCAT1 inhibitor) or GABA (used as a control). After 7 days of plating, spheres larger than 40 μm in diameter were scored to determine the sphere-forming efficiency. *n* = 3 for each condition. Data are presented as mean ± SD. One-way ANOVA with Tukey’s multiple-comparison test was performed. (E and F) Sphere-forming efficiency with BCAT suppression. MDA-MB-231 cells were transduced with lentiviral Dox-inducible shRNA against the BCAT gene. After 3 days of culture with or without Dox, (E) protein suppression by the shRNA was confirmed by immunoblot, and (F) sphere formation assays were performed as described above. *n* = 3 for each condition in the sphere formation assay. Data are presented as mean ± SD. One-way ANOVA with Tukey’s multiple-comparison test was performed. See also Figures S7A, S7B, S7C, S7D, and S7E. (G and H) Sphere-forming efficiency of basal-like breast cancer cells with BCAT1 overexpression. The basal-like HCC1143 cells were retrovirally transduced with MSCVneo/BCAT1 for BCAT1 overexpression. (G) BCAT1 protein expression was validated by immunoblot, and (H) sphere formation assay was performed. *n* = 3 for each condition in the sphere formation assay. Data are presented as mean ± SD. Unpaired two-tailed *t* test with Welch’s correction was performed. See also Figure S7F. Statistical significance is indicated by asterisks based on *p* values: *p <0.05, **p <0.01, ***p <0.005, and ****p <0.001. n.s., not significant.

In the mammalian BCAA metabolic pathway, BCAT1 (branched-chain amino acid transaminase 1, cytoplasmic) regulates cancer development and maintenance, and the enzyme is functionally essential for BCAA production and tumor-initiating cell activity in myeloid leukemia and NSCLC.^11,12^ We hypothesized that BCAT1 augments BCAAs in breast cancer cells and contributes to tumor growth. First, we analyzed the gene expression of *BCAT1* and its paralog *BCAT2*, a mitochondrial BCAA transaminase, using data from the Cancer Cell Line Encyclopedia (CCLE).^26–28^ *BCAT1* is upregulated in the claudin-low subtype compared with other subtypes, i.e., luminal, HER2-positive, or the basal-like subtype of TNBC cell lines (Figure 3A). On the other hand, *BCAT2* expression was downregulated rather than upregulated in the claudin-low subtype, compared with non-TNBC subtypes (Figure 3A). At the protein level, BCAT1 was notably higher in claudin-low MDA-MB-231 and Hs578T cells, while it was undetectable in MCF7 and BT-474 (luminal), MDA-MB-453 and SK-BR-3 (HER2-positive), and HCC1143 (basal-like) cells, as well as in MCF10A, a non-transformed breast epithelial cell line (Figure 3B). BCAT2 expression was lower in claudin-low subtypes than in luminal subtypes, indicating a reciprocal trend to BCAT1 in breast cancer cells (Figure 3B). We examined whether the transaminase activity of BCAT1 controls breast cancer cell growth. Gabapentin, a gamma-aminobutyric acid (GABA) and leucine analog, inhibits the transaminase activity of BCAT1 but not BCAT2.^29,30^ In the presence of gabapentin, sphere-forming ability was significantly reduced in the MDA-MB-231 and Hs578T CL-TNBC cells, whereas GABA had no effect on their sphere formation (Figures 3C and 3D).

To determine the requirement for BCAT1, we employed a doxycycline (Dox)-inducible short hairpin RNA (shRNA)-mediated gene knockdown approach. In MDA-MB-231 cells, Dox treatment led to approximately an 80% reduction of BCAT1 protein after 96 h and significantly suppressed sphere-forming ability, whereas BCAT2 knockdown showed no discernible impact on sphere formation (Figures 3E and 3F). Interestingly, in the luminal subtype MCF7 breast cancer cells, neither BCAT1 nor BCAT2 knockdown had a significant impact on sphere-forming ability (Figure S7A). Likewise, BCAT1 knockdown had no significant effect on the clonogenic growth of HER2-positive MDA-MB-453 and SK-BR-3 cells (Figure S7B). For genetic loss of BCAT1 and BCAT2, we used several independent constructs for shBCAT1 knockdown and sgBCAT2 knockout for sphere formation assays (Figures S7C, S7D, and S7E). The reduction in sphere-forming ability was well correlated with BCAT1 knockdown efficiencies (Figures S7C and S7D). BCAT2 knockouts, however, had no apparent impact on sphere formation, consistent with BCAT2 knockdown results described earlier (Figures S7C and S7E). Overexpression of BCAT1 in the basal-like HCC1143 cells significantly enhanced sphere-forming ability (Figures 3G and 3H). On the other hand, either BCAT1 or BCAT2 overexpression had a marginal effect on cell growth in luminal MCF7 cells (Figure S7F). BCAT1 overexpression in the sorted OLIVe FRET-Low population neither affected the OLIVe-FRET ratio nor sphere formation in MDA-MB-231 CL-TNBC cells (Figures S7G and S7H), likely due to the high expression of BCAT1 in CL-TNBC cells (Figure 3B). Collectively, these results demonstrate the functional requirement of BCAT1 and BCAA metabolism for CL-TNBC proliferation.

As upstream regulators of BCAT1 expression in CL-TNBC, we examined the transcription factor c-MYC and the RNA-binding protein MSI, both of which have been shown to regulate BCAT1.^11,31^ c-MYC expression was higher in basal-like HCC1143 and in claudin-low MDA-MB-231 and Hs578T cells (Figure S8A). CRISPR-mediated knockout of c-MYC reduced BCAT1 expression by about 20%–30% in MDA-MB-231 cells (Figure S8B), suggesting that c-MYC partially contributes to BCAT1 upregulation in CL-TNBC. We found that MSI1 and MSI2 were highly expressed in the luminal subtype MCF7 and BT-474 cell lines, whereas these genes were only marginally expressed in other cell lines (Figure S8A). Functionally, overexpression of MSI1 or MSI2 did not alter BCAT1 levels in MCF7 cells (Figure S8C). By contrast, in Hs578T CL-TNBC cells, overexpression of MSI1 or MSI2 increased BCAT1 protein expression by 50% or 36%, respectively, indicating a positive role for MSI1/2 in regulating BCAT1 expression in CL-TNBC cells.

### BCAT1 mediates BCAA production in CL-TNBC cells

**Figure 4.**
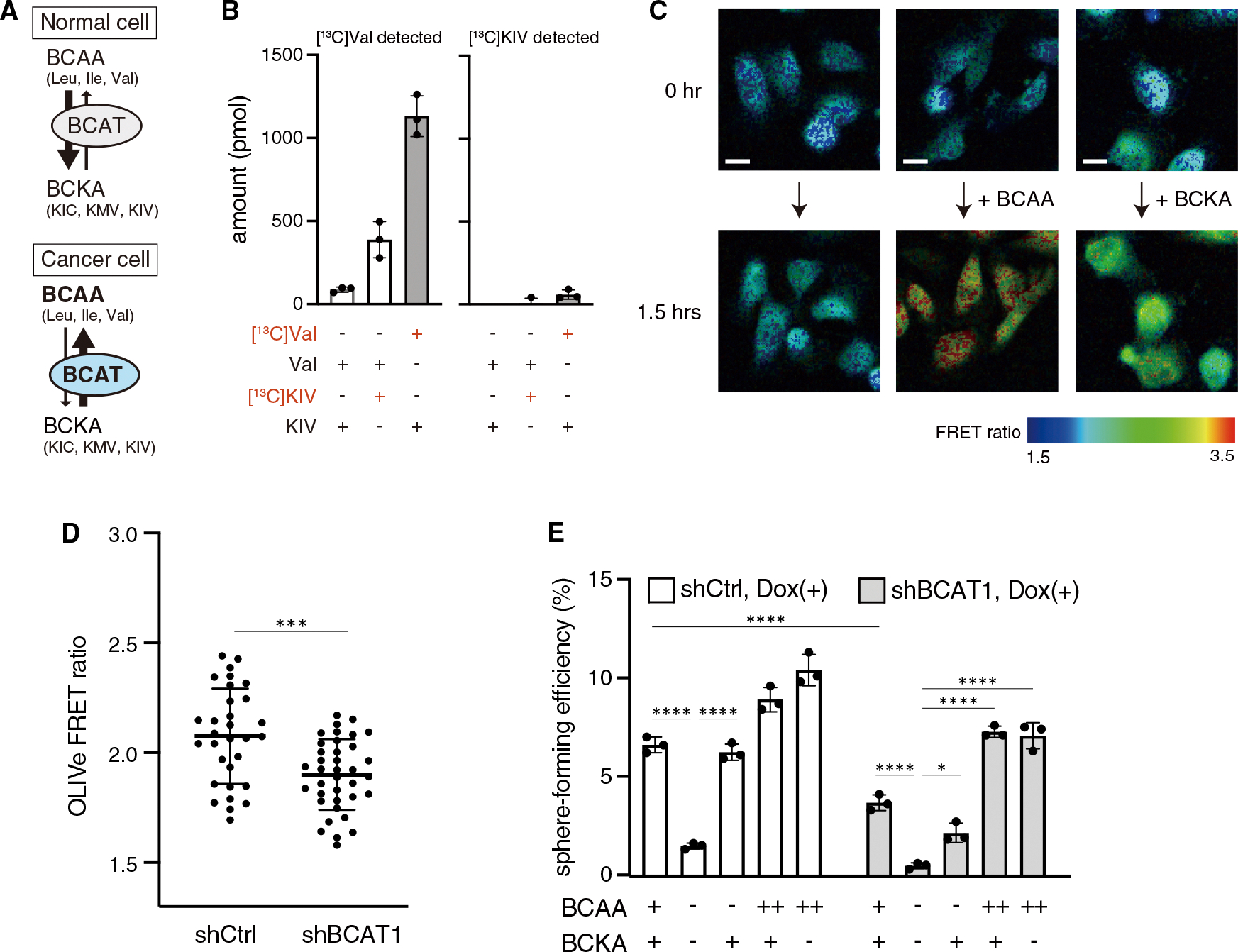
BCAT1 mediates BCAA production in claudin-low TNBC cells (A) BCAA metabolism in normal and cancer cells. In normal cells, catabolic oxidation of BCAAs to BCKAs predominates, whereas in cancer cells, anabolic BCAA production through transamination of BCKAs is increased by BCAT1 activation. (B) Fates of BCAA and BCKA in CL-TNBC cells. MDA-MB-231 cells were cultured with ^13^C-labeled valine and unlabeled KIV or with ^13^C-labeled KIV and unlabeled valine for 30 min. Metabolites were extracted with 80% methanol, vacuum-dried, and analyzed for ^13^C-incorporated valine or KIV measurements using ^1^H-^13^C HSQC NMR. Data are presented as mean ± SD. *n* = 3 for each condition. See also Figures S9A, S9B, and S10A. (C) BCAA production from BCKA in CL-TNBC cells at single-cell resolution. MDA-MB-231 cells with stable OLIVe expression were cultured for 1.5 h after BCAA removal. Subsequently, BCAAs or BCKAs were added back at concentrations matching those in the standard RPMI 1640 media formulation: valine or KIV at 170 μM and leucine, KIC, isoleucine, or KMV at 381 μM. OLIVe FRET signals were monitored through time-lapse imaging at 10 min intervals, and the FRET ratio after 1.5 h was shown as pseudocolor. FRET ratio values were presented as a graph in Figures S5C, S5D, and S9C. Scale bars represent 20 μm. (D) OLIVe FRET ratio following BCAT1 knockdown. Dox-inducible shCtrl or shBCAT1 was introduced via lentiviral transduction into MDA-MB-231 cells that stably express OLIVe. After puromycin selection of transduced cells, shRNA was induced with Dox for 3 days. Cells were seeded in 0.5% methylcellulose medium containing BCKAs but lacking BCAAs. The OLIVe FRET signal was detected using the AxioObserver7 microscope, and the FRET ratio was calculated from the acquired images. The number of cells analyzed was *n* = 31 and *n* = 37 for shCtrl and shBCAT1, respectively. Data are presented as mean ± SD. Unpaired two-tailed *t* test with Welch’s correction was used for statistical analysis. See also Figure S9D. (E) Sphere formation with BCAT1 knockdown, with or without BCAA or BCKA. MDA-MB-231 cells with Dox-inducible shCtrl or shBCAT1 were cultured with Dox for 3 days; then, cells were seeded in 0.5% methylcellulose medium with either BCAA or BCKA as indicated. “+” for BCAAs indicates the concentration used in standard RPMI 1640 medium as follows: leucine 381 μM, isoleucine 381 μM, and valine 170 μM. “++” for BCAAs indicates an excess amount of BCAAs at 2 mM each for leucine, isoleucine, and valine. “+” for BCKAs indicates 30 μM for each KIC, KMV, and KIV. After 7 days of culture, spheres larger than 40 μm in diameter were counted. Results are shown as sphere-forming efficiency based on the seeded cell number. *n* = 3 for each group. Data are presented as mean ± SD. One-way ANOVA with Tukey’s multiple-comparison test was performed. See also Figures S10B and S10C. Statistical significance is indicated by asterisks based on *p* values: *p <0.05, ***p <0.005, and ****p <0.001.

In myeloid leukemia, BCAT1 promotes BCAA production by catalyzing the transamination of BCKA (Figure 4A).^11,32^ To examine whether CL-TNBC cells also produce BCAA from BCKA, we analyzed the metabolic fates of valine and its ketoacid precursor alpha-keto-isovalerate (KIV) using ^1^H-^13^C heteronuclear single quantum correlation (HSQC), an NMR spectroscopy technique that specifically detects ^13^C-labeled compounds.^33^ MDA-MB-231 or Hs578T CL-TNBC cells were cultured in the presence of ^13^C-labeled valine or KIV, and HSQC spectra were collected using the cell extracts prepared at 30 min after the addition of a ^13^C-labeled substrate. When ^13^C-labeled valine was used, we included unlabeled KIV in the medium, and vice versa, to keep valine and KIV concentrations equal across experiments. When cells were cultured with ^13^C-KIV, we observed the appearance of ^13^C-valine, indicating rapid intracellular valine generation via KIV transamination (open bars in Figure 4B; Figures S9A and S9B for MDA-MB-231 and Hs578T cells, respectively). Concentrations of glutamine, a precursor for glutamate, which is a co-substrate for BCAT1-dependent transamination reactions, did not affect the conversion rates between KIV and valine, indicating that glutamate concentration is not a rate-limiting factor in the transamination of intracellular KIV (Figures S9A and S9B). On the other hand, when ^13^C-valine was added, ^13^C-KIV was barely detectable, despite its efficient import into the cells, indicating no significant intracellular deamination of valine to KIV (closed bars in Figure 4B; Figures S9A and S9B). These results indicate that BCAA production from BCKA, rather than BCAA oxidation, is predominant in CL-TNBC cells.

We confirmed intracellular BCAA production using OLIVe-expressing MDA-MB-231 CL-TNBC cells. BCAA production from BCKAs was monitored at the single-cell level. The FRET ratio decreased from approximately 3.0 to 2.0 upon BCAA removal from the culture medium (Figures 4C and S9C). The OLIVe FRET ratio increased from 2.0 to 3.0 or 2.4 when BCAAs or BCKAs were replenished, respectively (Figures 4C and S9C). To determine whether BCAT1 is required for the conversion of BCKAs to BCAAs in CL-TNBC cells, we measured the OLIVe FRET ratio after shRNA-mediated knockdown of BCAT1 under suspension culture conditions (Figures 4D and S9D). BCAT1 knockdown attenuated the recovery of the FRET ratio in the presence of BCKAs but not BCAAs, suggesting that *in situ* BCAA production is BCAT1 dependent.

Next, we examined whether intracellular BCAA production is functionally essential for CL-TNBC growth. We found that BCAA removal from culture media dramatically inhibited sphere formation in HER2-positive MDA-MB-453 and SK-BR-3 cells, as well as in CL-TNBC MDA-MB-231 and Hs578T cells (Figure 1B). Adding the three BCKAs to BCAA-depleted media completely rescued the sphere-forming ability of CL-TNBC cells, comparable to that in the standard BCAA-containing media, whereas the rescue was incomplete in HER2-positive cells (Figures 1B and 4E). Interestingly, BCAA depletion had less impact on luminal and basal-like breast cancer subtypes than on claudin-low subtypes, suggesting that the dependency on the BCAT1-BCAA pathway is specific to CL-TNBC cells (Figure 1B).

Using operando NMR spectroscopy, we monitored the conversion of ^13^C-labeled KIV to ^13^C-labeled valine in live cells in real time (Figure S10A). In CL-TNBC MDA-MB-231 and Hs578T cells, which rely heavily on BCAAs and BCKAs for growth, we observed rapid KIV-to-Val conversion, detectable only after 16 min (cycle 8) from the start of metabolite tracing. In contrast, in luminal MCF7 and BT-474 cells, which do not depend on BCAAs, the conversion of KIV to Val was barely detectable even after 40 min at the assay endpoint. These results suggest that CL-TNBC cells have an enhanced ability to produce BCAAs from BCKAs. Interestingly, in HER2-positive subtypes, KIV-to-Val conversion was detected in SK-BR-3 cells but not in MDA-MB-453 cells. These findings align well with our sphere formation assay results under BCAA-depleted and BCKA-present conditions (Figure 1B). MDA-MB-453 cells did not proliferate in the presence of BCKAs, whereas SK-BR-3 cells were able to grow with BCKAs. Notably, in basal-like HCC1143 cells, KIV-to-Val conversion occurred at a rate comparable to that in CL-TNBC cells. Because HCC1143 cells are not dependent on BCAAs and BCKAs for proliferation (Figure 1B), the cellular ability to produce BCAAs does not appear to be a critical factor for their proliferation.

Functionally, CL-TNBC cells with BCAT1 knockdown showed reduced sphere-forming ability even in the presence of BCKAs. Furthermore, adding excess BCAAs (5-fold more for Leu and Ile and 11-fold more for Val) rescued the reduced sphere-forming ability caused by BCAT1 knockdown (Figure 4E). To confirm the requirement for BCAT1 transaminase activity in CL-TNBC cell proliferation, we used a transamination-defective BCAT1 mutant, K222A, which is unable to produce BCAAs from BCKAs (Figures S10B and S10C). Overexpression of wild-type BCAT1 rescued the defective sphere-forming ability caused by BCAT1 knockdown, but expression of the K222A mutant did not, indicating that BCAT1 transaminase activity is necessary for clonogenic growth in CL-TNBC. Together, these results indicate that BCAAs are generated intracellularly from their precursor BCKA in a BCAT1-dependent manner and that BCAT1 plays a pivotal role in claudin-low breast cancer cell proliferation.

### Activation of the BCAT1-BCAA axis in primary human TNBC

**Figure 5.**
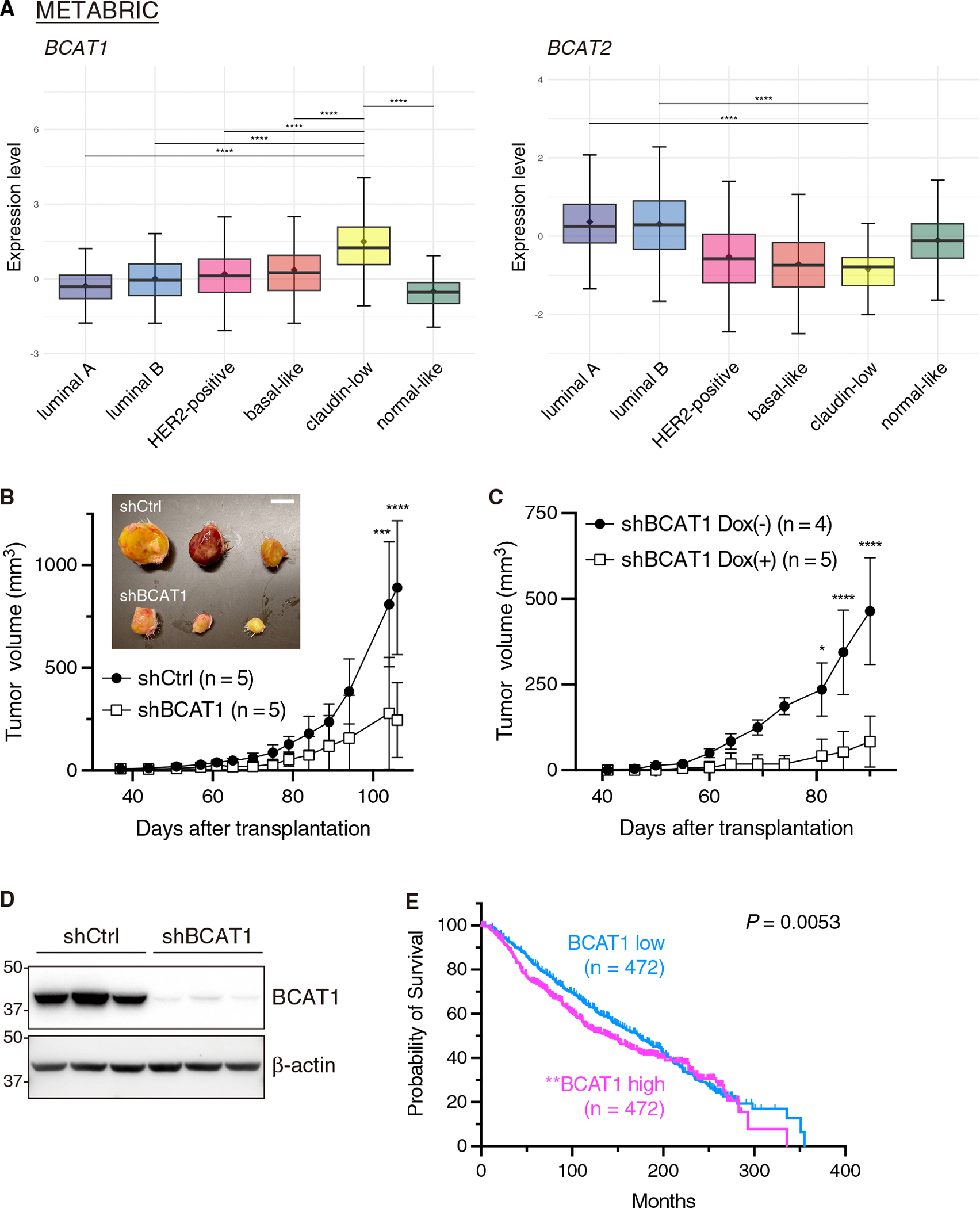
Activation of the BCAT1-BCAA axis in primary human TNBC (A) Analysis of BCAT expression in patients with breast cancer using the METABRIC dataset. Results are organized by breast cancer subtypes: luminal A (*n* = 686), luminal B (*n* = 467), HER2-positive (*n* = 231), basal-like (*n* = 269), claudin-low (*n* = 79), and normal-like (*n* = 155). Data are shown as boxplots with the median (center line) and mean (black diamond). Whiskers represent mean ± 2 × SD. Welch’s *t* test was used for pairwise comparisons between claudin-low and each of the other subtypes. See also Figure S11A. (B, C, and D) Xenograft tumor formation with BCAT1 suppression. (B and C) MDA-MB-231 cells with stable OLIVe expression and Dox-inducible shCtrl or shBCAT1 were orthotopically transplanted into the mammary fat pads of NSG recipients. Doxycycline was given daily by oral gavage for the first 7 days after transplantation to induce shRNA expression. Thereafter, mice were fed chow containing 0.03% doxycycline. Tumor volume was measured periodically until sacrifice. *n* = 5 for both shCtrl and shBCAT1 groups in (B). *n* = 4 for the Dox(−) group and *n* = 5 for the Dox(+) group in (C). Tumor volumes are shown as mean values with SEM. Two-way ANOVA with Sidak’s multiple-comparison test was performed. Upon sacrifice, tumors were dissected (B, inset; scale bars represent 10 mm)), and (D) protein lysates were prepared for BCAT1 expression analysis by immunoblot. β-actin served as a loading control. See also Figures S12A and S12B. (E) Kaplan-Meier survival analysis of breast cancer patients using the METABRIC dataset. Patients were divided into two groups based on BCAT1 expression levels. The top and bottom quartiles were classified as BCAT1 high and BCAT1 low, respectively. *n* = 472 per cohort. The Gehan-Breslow-Wilcoxon test was performed for statistical analysis. *p* = 0.0053. Statistical significance is indicated by asterisks based on *p* values: *p <0.05, **p <0.01, ***p <0.005, and ****p <0.001.

We examined BCAT1 expression in human breast cancer patients using datasets from the METABRIC and The Cancer Genome Atlas (TCGA) databases.^34–36^ In the METABRIC data, BCAT1 expression varies across subtypes and is significantly higher in the claudin-low type than in normal-like, luminal A/B, HER2-positive, or basal-like (Figure 5A, left), consistent with our findings in breast cancer cell lines (Figures 3A and 3B). Analysis of the TCGA dataset supported these results; BCAT1 expression was significantly higher in claudin-low subtypes (Figure S11A). In contrast, BCAT2 expression levels were lower in claudin-low compared to other subtypes, particularly luminal A/B (Figures 5A, right, and S11A). Next, we tested whether BCAT1-dependent BCAA metabolism contributes to cancer aggressiveness *in vivo*. MDA-MB-231 cells stably expressing OLIVe with Dox-inducible shCtrl or shBCAT1 were orthotopically transplanted into immunodeficient mice, and tumor formation was monitored (Figures 5B, 5D, and S12A). Dox-inducible shBCAT1 with or without Dox was also compared for tumor formation (Figure 5C). BCAT1 knockdown resulted in significantly smaller tumors than control knockdown in both experimental designs, indicating that BCAT1 is essential for CL-TNBC proliferation *in vivo*. We measured BCAA concentrations in the peripheral blood from mice bearing either shCtrl or shBCAT1 tumors and found no difference in BCAA levels between the groups (Figure S12B). Although intracellular BCAA levels are regulated by BCAT1 (Figure 4D), extracellular BCAA levels *in vivo* do not seem to depend solely on BCAT1. In breast cancer patients, Kaplan-Meier analysis of the METABRIC dataset indicates that higher BCAT1 expression predicts poorer prognosis (Figure 5E). Together, these results support our hypothesis that BCAT1 regulates tumor aggressiveness in human CL-TNBC.

### Activated BCAA metabolism due to reduced BCKDH complex expression in TNBC

**Figure 6.**
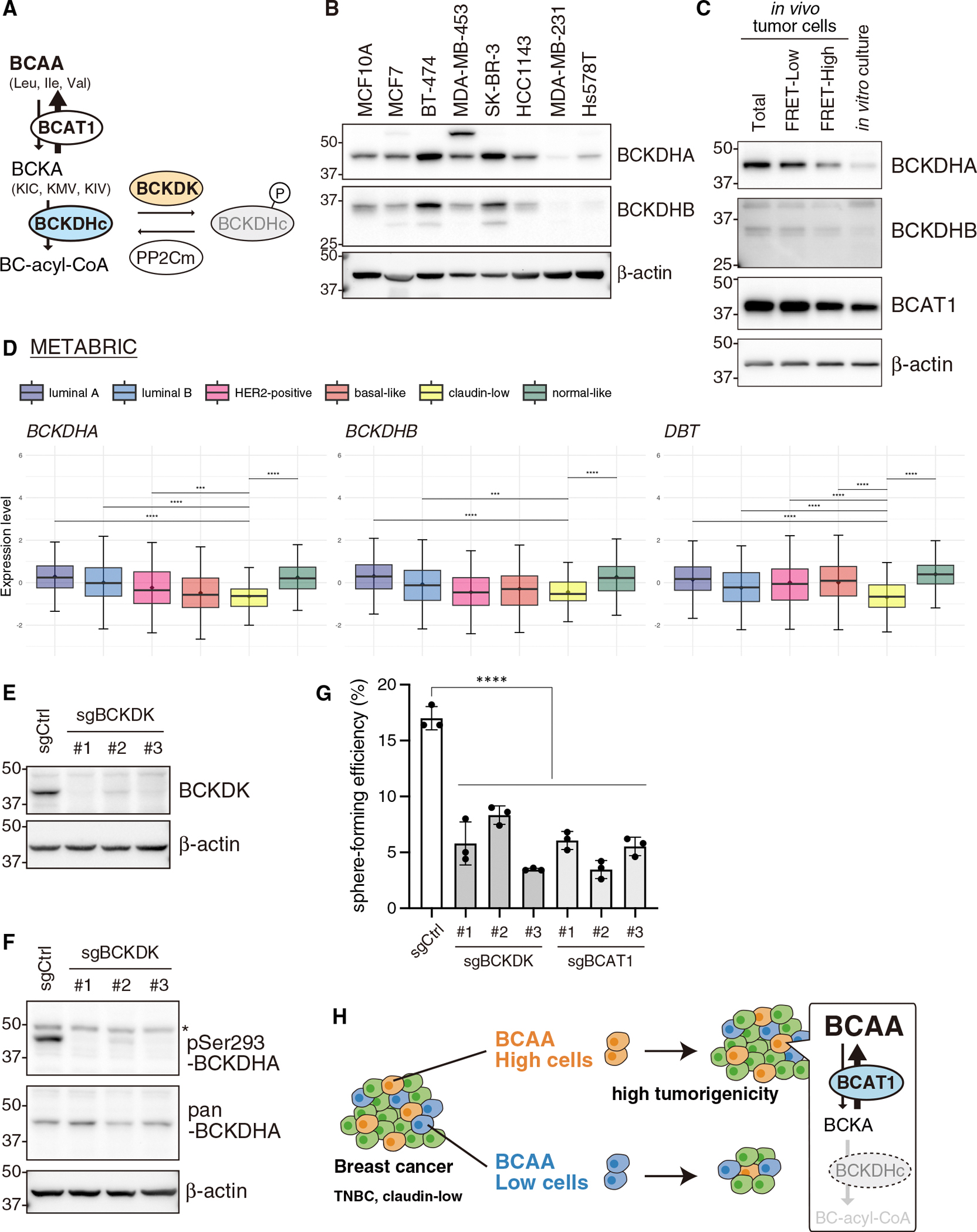
Activated BCAA metabolism due to reduced BCKDH complex expression in TNBC (A) Schematic of BCAA metabolism, including the BCKDH complex (BCKDHc) and BCKDK. In BCAA metabolism, the interconversion of BCAAs and BCKAs is mediated by the enzyme BCAT. In some cancer cell types, BCKA-to-BCAA conversion by BCAT1 is predominant. BCKDHc irreversibly converts BCKAs into BC-acyl-CoAs. BCKDHc activity is regulated by inhibitory phosphorylation of BCKDHA at Ser293. BCKDK and PP2Cm are the kinase and phosphatase regulating BCKDHA inhibitory phosphorylation, respectively. (B and C) (B) Expression of BCKDHc subunits in breast cancer cells, and (C) in OLIVe FRET-High or -Low cells sorted from a xenograft tumor. β-actin served as the loading control. See also Figures S14A, A14B, S15A, and S15B. (D) Expression of BCKDHc subunit genes in breast cancer patients from the METABRIC dataset. Results are organized by breast cancer subtypes as indicated: luminal A (*n* = 686), luminal B (*n* = 467), HER2-positive (*n* = 231), basal-like (*n* = 269), claudin-low (*n* = 79), and normal-like (*n* = 155). Data are shown as boxplots with the median (center line) and mean (black diamond). Whiskers represent mean ±2 × SD. Welch’s *t* test was used for pairwise comparisons between claudin-low and each of the other subtypes. See also Figure S11B. (E, F, and G) BCKDHc reactivation following BCKDK knockout and clonogenic growth of CL-TNBC MDA-MB-231 cells. BCKDK expression was suppressed by lentiviral sgRNA transduction. Three distinct sgRNA targeting sequences were used. (E) BCKDK knockout efficiency was validated by immunoblot. (F) The phosphorylation status of BCKDHA at Ser293 and total BCKDHA expression levels were analyzed by immunoblot. The asterisk indicates a non-specific band. (G) BCKDK knockout cells were seeded for sphere formation assays in a 0.5% methylcellulose suspension condition. After 7 days of culture, spheres were counted, and sphere-forming efficiency was calculated. *n* = 3 for each condition in sphere formation assays. Data are presented as mean ± SD. One-way ANOVA with Tukey’s multiple-comparison test was performed. (H) Graphical abstract of this study. Statistical significance is indicated by asterisks based on *p* values: ***p <0.005 and ****p <0.001.

While we demonstrated the functional requirement for BCAT1 in BCAA metabolism in CL-TNBC cells, upregulation of BCAT1 alone does not fully account for heterogeneity in BCAA levels within tumor tissue. Indeed, we observed no significant differences in the uptake of neutral amino acids between OLIVe FRET-Low and -High cells (Figure S13). Therefore, we sought potential regulators that could contribute to the heterogeneity in intracellular BCAA levels and metabolism. The BCKDHc, consisting of the BCKDHA, BCKDHB, DBT, and DLD subunits, catalyzes the irreversible decarboxylation of BCKAs to generate the corresponding BC-acyl-CoAs (Figure 6A). We hypothesized that BCKDHc activity may contribute to the differences in BCAA levels in breast cancer cells. Interestingly, expression of the BCKDHA and BCKDHB subunits was apparently lower in CL-TNBC cells than in other breast cancer subtypes (Figure 6B). Notably, this expression pattern was conserved in tumors formed *in vivo*. Levels of BCKDHc subunits were markedly lower in OLIVe FRET-High cells than in FRET-Low cells (Figure 6C). In contrast, BCAT1 expression did not show the same pattern in FRET-High and -Low cells (Figure 6C). While BCAA supplementation increased the FRET ratio and enhanced sphere formation in OLIVe FRET-Low cells, BCKA supplementation neither increased the FRET ratio nor sphere formation (Figures 2F and 2G), suggesting that BCKA-to-BCAA conversion is attenuated in FRET-Low cells. Importantly, claudin-low breast cancer patients exhibited the lowest levels of BCKDHA and BCKDHB expression compared with luminal A/B and normal-like subtypes (Figure 6D). In addition, analysis of the METABRIC and TCGA datasets revealed downregulation of DBT, an essential subunit of BCKDHc (Figures 6D and S11B).

To obtain further supporting evidence, we examined the impact of genetic loss of BCKDK on CL-TNBC proliferation. BCKDK is a Ser/Thr kinase for the BCKDHA subunit, and the phosphorylation of BCKDHA at the Ser293 residue inhibits BCKDHc enzymatic activity. We used lentiviral transduction to deliver Cas9 and single-guide RNAs (sgRNAs) targeting BCKDK. Knockout efficiency was approximately 90% (Figure 6E), and phosphorylation of BCKDHA at Ser293 decreased in parallel with knockout efficiency across three target sequences (Figure 6F). BCKDK knockout in MDA-MB-231 cells reduced their sphere-forming potential in proportion to knockout efficiency, to levels comparable to those observed with BCAT1 knockout (Figure 6G).

To better understand the regulatory mechanisms governing intracellular BCAA levels and cancer stem cell (CSC) maintenance, we conducted transcriptome analysis of FRET-High and -Low cells purified from xenograft tumors via RNA sequencing (RNA-seq) (Figures S14A and S14B). Gene set enrichment analysis of the differentially expressed genes identified several pathways that were differentially regulated between the two populations (Figure S14A). In FRET-High cells, upregulated gene sets include “E2F targets,” which enhance cell cycle progression (Figures S14A and S14B). Downregulated gene sets in FRET-High (i.e., upregulated in FRET-Low) include “glycolysis” and “TNF-α signaling via NF-κB.” Alterations in these pathways potentially regulate CSCs via BCAA metabolism (Figures S14A and S14B).

Collectively, these results indicate that the balance between BCAA synthesis and oxidative breakdown controls intracellular BCAA levels and CL-TNBC growth. Coupled with BCAT1 upregulation, this metabolic reprogramming is a determinant of cellular BCAA levels, tumor heterogeneity, and breast CSCs in this human malignancy (Figure 6H).

## DISCUSSION

In this study, we investigated how BCAA metabolism regulates tumor growth in breast cancer. Our study reveals that cellular BCAA levels and dependence determine proliferative potential. Interestingly, BCAA dependence varies across breast cancer subtypes: CL-TNBC and HER2(+) are highly dependent on BCAAs, whereas other subtypes are not. We also demonstrated that breast cancer cells with elevated BCAAs exhibit higher tumor-initiating potential and identified molecular mechanisms that regulate cellular BCAA concentration and tumor heterogeneity.

### Subtype-specific dependency on BCAA metabolism in human breast cancer

Previous studies have reported functional dependencies on BCAA metabolism in several types of cancer, including myeloid leukemia, glioma, NSCLC, PDAC, and breast cancer.^11–13,15–17,32,37^ In breast cancer, identifying the subtype has a major clinical impact on treatment strategies and can be guided by gene expression patterns.^34,38–40^ Our study unveils subtype-specific requirements for BCAA metabolism; CL-TNBCs, unlike other breast cancer subtypes, are highly dependent on BCAAs. Interestingly, these CL-TNBC cells do not exhibit enhanced uptake of extracellular BCAAs; instead, BCAA dependency arises from the need for intracellular BCAA biosynthesis via BCKA transamination. Because the fate of BCAAs in CL-TNBC may regulate tumor-initiating potential, it is important to understand their metabolic fates within tumor cells. BCAA activation of the mTOR pathway contributes to cancer cell proliferation in myeloid leukemia.^11,32^ However, in CL-TNBC cells, mTORC1 activation was not markedly higher in comparison to other breast cancer subtypes and was even lower than in luminal breast cancer cells, as measured by S6K phosphorylation (Figure S15A). 4E-BP phosphorylation, another downstream readout of mTORC1 activity, showed no apparent differences across subtypes (Figure S15A). Consistently, suppression of BCAT1 expression did not affect mTORC1 activation in CL-TNBC (Figure S15B).

Each human breast cancer subtype preferentially uses either aerobic or anaerobic respiration for growth, and the metabolic shift from oxidative phosphorylation to glycolysis is known to drive chemotherapeutic resistance in human TNBC.^41,42^ These metabolic pathways are reported to regulate dormancy or stem cell potential in human breast cancers.^43^ In this study, we identified BCAA metabolism as another regulatory mechanism of subtype specificity and stem cell properties in breast cancer. It implies that BCAA metabolism regulates the subtype-specific metabolic preferences previously reported.^41,42^ The claudin-low subtype is often classified within TNBC and considered the most aggressive type,^40,44^ although its classification remains controversial, since some tumors with a claudin-low phenotype are not always TNBCs.^45,46^ Claudin-low breast cancer cells express low levels of claudins, which contribute to cell-to-cell junctions, and high levels of mesenchymal and stemness genes, such as vimentin, Snail, and Slug. In addition, BCAA metabolic enzyme expression is upregulated in recurrent breast cancer patients,^16^ and gene expression profiles in breast cancer cells after conventional chemotherapy display profiles similar to those of the claudin-low subtype.^39^ These previous findings suggest that CL-TNBC patients could benefit from combination therapies specifically targeting BCAA metabolic pathways.

### BCAA metabolism regulates CSC potential

CSCs are defined as cells capable of initiating tumors.^23,47^ CSCs are often found as small subpopulations within tumor tissue. These cells possess self-renewal capacity and form the basis for cellular heterogeneity. We found that cellular BCAA levels in TNBC cells are highly heterogeneous within tumors, and the BCAA-High population exhibits a higher probability of containing cells with tumor-initiating potential, i.e., CSCs. Using *in vivo* limiting dilution assays, we provide further evidence that the BCAA-High population contains more CSCs than the BCAA-Low population (Figure 2E), suggesting that intracellular BCAA concentration potentially regulates CSC functions *in vivo*.

Previous studies have identified CD44^+^/CD24^−/low^ cells as a population enriched for tumor-initiating capacity in human breast cancer.^23,48^ Our results suggest that intracellular BCAA concentration is a biomarker of CSCs, alongside the well-established roles of CD44 and CD24 in breast cancer. Indeed, CSCs defined by the CD44^+^/CD24^−/low^ phenotype were more enriched in FRET-High cells than in FRET-Low cells in CL-TNBC MDA-MB-231 and Hs578T cells, indicating a positive correlation with intracellular BCAA levels (Figure S16A). ALDH activity, another potential CSC marker, did not directly correlate with the OLIVe-FRET ratio in MDA-MB-231 cells, whether cultured *in vitro* or isolated from xenograft tumors *in vivo*, as detected by the recently developed ALDH probe C5S-A^49^ (Figures S16B and S16C). These results suggest that cellular BCAA levels and ALDH activity can serve as independent markers for CSCs, at least in CL-TNBC.

RNA-seq analysis identified several pathways altered in the BCAA-High and -Low populations. In BCAA-High cells, upregulated gene sets include “E2F targets,” indicating active cell cycle progression and a growth advantage for the BCAA-High population. On the other hand, downregulated gene sets in the BCAA-High population include glycolysis and TNF-α signaling via NF-κB. Several recent studies have shown that reduced glycolytic activity and activated oxidative phosphorylation are key hallmarks of CSCs.^50–52^ Therefore, the downregulation of glycolysis in BCAA-High aligns with our findings that tumor-initiating CSCs are more abundant in this population. Interestingly, a previous study demonstrated that activation of the TNF-α signaling pathway in non-CSCs contributes to stem cell maintenance in basal-like TNBC.^53^ It is thus plausible that upregulation of TNF-α signaling in the BCAA-Low population contributes to the maintenance and survival of CSCs in the BCAA-High population in CL-TNBC as well.

### Elevated intracellular BCAAs are maintained by altered expression of BCAA metabolic enzymes

Traditional methods of metabolite analysis, such as capillary electrophoresis-mass spectrometry or fluorescent derivatization with HPLC, require cell lysis and, in most cases, more than 100,000 cells to reliably and reproducibly quantify metabolite levels. Thus, it was impossible to examine the phenotype-metabolite relationship in live cells at the single-cell level. In this study, we analyzed the roles of BCAAs in regulating the behavior of individual cancer cells by employing a genetically encoded BCAA biosensor. We show that cells with high BCAA levels have increased tumor-initiating potential through a unique mechanism that controls intracellular BCAA levels. Our OLIVe FRET-based cell sorting and transplantation assay indicated that tumor-initiating cells are enriched in a cell population with higher BCAA concentrations. The heterogeneity in intracellular BCAA levels is independent of differences in amino acid uptake in CL-TNBC tumors, since amino acid import activities were similar between OLIVe FRET-High and -Low cells (Figure S13). Instead, in CL-TNBC cells, downregulation of the BCKDHc appears to help maintain high BCAA levels. Previous reports demonstrated that the expression of BCKDHc components is transcriptionally upregulated in PDAC.^37^ In our study, BCKDHc components are transcriptionally downregulated in CL-TNBC patients compared with other subtypes, indicating that the regulation of BCKDHc expression seems to be cancer type dependent. Our analysis indicates that BCAT1 upregulation and BCKDHc downregulation contribute to the maintenance of an elevated intracellular BCAA pool in CL-TNBC. The gene expression signatures in the claudin-low subtype are closely associated with those of tumor-initiating ability and epithelial-mesenchymal transition.^39,40^ Therefore, suppressed BCAA catabolism is a driver of malignant properties in human breast cancer. Because BCKDHc reactivation by BCKDK suppression reduced CL-TNBC cell proliferation (Figures 6E, 6F, and 6G), BCKDK inhibitors, such as BT2, could serve as an effective therapeutic strategy for CL-TNBC when combined with a BCAT1 inhibitor.

### Limitations of the study

While our results provide molecular and functional evidence for the regulatory role of BCAA metabolism in TNBC, several limitations remain. First, most experiments were performed using CL-TNBC cell lines. Real-time detection of intracellular BCAA levels in primary human TNBC cells, along with functional characterization of the BCAA-High and BCAA-Low populations, would further support the clinical relevance of our findings. Second, it remains unclear how intracellular BCAA levels regulate CSC potential in CL-TNBC. Because suppression of BCKA catabolism, in addition to that of BCAA itself, is required to maintain the BCAA-High population, identifying which downstream metabolic species regulate CSC potential will be an important next step. Third, our *in vivo* imaging of intracellular BCAA, relying on intensity-based FRET ratio calculations, did not allow quantitative comparisons between individual mice or across different time points because the FRET ratio is influenced by laser power fluctuations and background signals in two-photon microscopy. With fluorescence lifetime imaging microscopy (FLIM), we can perform absolute quantification of intracellular BCAA *in vivo* because FLIM relies on an intrinsic fluorescence property that is not affected by fluctuating experimental parameters.^54^ Therefore, developing an FLIM-optimized BCAA biosensor would help us understand how this metabolic pathway regulates the maintenance of CSCs in human TNBC.

## RESOURCE AVAILABILITY

### Lead contact

Further information and requests for resources and reagents should be directed to and will be fulfilled by the lead contact, Takahiro Ito (takahiro.ito@infront.kyoto-u.ac.jp).

### Materials availability

This study did not generate new unique reagents.

### Data and code availability

- RNA sequencing data have been deposited in the DDBJ BioProject: PRJDB42235^55,56^ (Run: DRR1048725-DRR1048730) and are publicly available as of the date of publication.
- NMR data have been deposited in the NIH Common Fund’s National Metabolomics Data Repository (NMDR), Metabolomics Workbench: PR003139. The data can be accessed via the project (https://doi.org/10.21228/M8XS06).
- This paper does not report original code.
- Any additional information required to reanalyze the data reported in this paper is available from the lead contact upon request.

## STAR★METHODS

Detailed methods are provided in the online version of this paper and include the following:

### EXPERIMENTAL MODEL AND STUDY PARTICIPANT DETAILS

#### Animal studies

All NOD.Cg-*Prkdc*^*scid*^ *Il2rg*^*tm1Wjl*^/SzJ (NSG) female mice were purchased from Jackson Laboratory via Oriental Bio Service and used for transplantation at 7 weeks of age unless otherwise indicated. All experimental procedures involving animals were approved by the Animal Experimentation Committee of Kyoto University (Approval No. P-21-4) and were conducted in accordance with the regulations for animal experiments at Kyoto University.

#### Cell lines

MCF7 (ID# RCB1904) and MDA-MB-453 (ID# 1192) cells were obtained from RIKEN BRC; HCC1143, MDA-MB-231, and MCF10A cells were kindly provided by Drs. Noriko Gotoh (Kanazawa University), Hisashi Kato (Osaka University),^58^ and Yasuyuki Fujita (Kyoto University), respectively. BT-474, SK-BR-3, and Hs578 T cells were from in-house stocks maintained at Chiba University. All cell lines were authenticated via STR profiling using the GenePrint 10 System (Promega) by BEX (Japan). Cells were routinely tested for mycoplasma contamination with the MycoAlert assay kit (Lonza) and were found to be negative.

#### Human datasets

Gene expression and clinical data from human breast cancer patients were obtained from two publicly available datasets: METABRIC (Molecular Taxonomy of Breast Cancer International Consortium) and TCGA-BRCA (The Cancer Genome Atlas - Breast Cancer cohort). For METABRIC, of the 1,980 available patient data, we used data from 1,887 samples in the analysis because clinical data were missing for the remaining 93 samples. The final METABRIC cohort comprised the following molecular subtypes: luminal A (*n* = 686), luminal B (*n* = 467), HER2-positive (*n* = 231), basal-like (*n* = 269), claudin-low (*n* = 79), and normal-like (*n* = 155). For TCGA-BRCA, gene expression and clinical data were available for 1,082 samples. Of these, 97 samples were excluded from our analysis due to missing subtype information. The final TCGA cohort comprised 985 samples with the following molecular subtypes: luminal A (*n* = 491), luminal B (*n* = 195), HER2-positive (*n* = 77), basal-like (*n* = 162), claudin-low (*n* = 32), and normal-like (*n* = 28). All data had already been de-identified in the original publications, and no new patient recruitment or sample collection was performed in this study. Patient information is available in the original publications.^34–36^

### METHOD DETAILS

#### DNA constructs

The pMSCV-Blast-OLIVe plasmid for the optical biosensor for leucine–isoleucine–valine (OLIVe) has been described previously.^19^ For cloning into Tol2-based vectors, the OLIVe coding region was amplified with Tks Gflex DNA Polymerase (Takara, cat# R060A) and inserted into EcoRI- and SalI-digested pT2ADW-6011NES vector (kindly provided by Dr. Michiyuki Matsuda, Kyoto University) to construct pT2ADW/OLIVe. The original pT2ADW-6011NES vector contains the D4Z4 insulator element^64^ (kindly provided by Dr. Akitsu Hotta, Kyoto University). The XE cocktail system was used for seamless DNA cloning.^65^ For transfection, X-tremeGENE 9 DNA Transfection Reagent (Thermo, cat# 6365787001) was used.

For the doxycycline-inducible shRNA gene suppression system, Teton-pLKO-puro plasmid was used. The plasmid was digested with AgeI and EcoRI, and annealed oligo DNA with the targeting sequence was inserted with DNA Ligation Kit Mighty Mix (Takara, cat# 6023).

For human BCAT1 overexpression, a lentiviral transduction system with the FG12-Ubc-hCD2-hBCAT1 transfer plasmid was used as previously described.^11^ For the aminotransferase activity mutant, a lentiviral construct that introduced the K222A point mutation was used.

For CRISPR-mediated gene knockout, lentiCRISPRv2 was used (a gift from Dr. Feng Zhang; Addgene plasmid #52961).^66^ The plasmid was digested with BsmBI, and the annealed oligo DNA with the guide RNA sequence was inserted by ligation.

#### Immunoblot

Cell lysates were prepared using RIPA lysis buffer (1% NP-40, 0.1% SDS, 0.5% sodium deoxycholate, 150 mM NaCl, 1 mM EDTA, 50 mM Tris-HCl pH7.4). After SDS-PAGE, proteins were transferred to a PVDF membrane (Millipore) using either a semi-dry electroblotting system (Atto) or a wet electroblotting system (Bio-Rad). The membrane was blocked with 0.5% skim milk in TBS-T. Blots were incubated with the primary antibodies, listed in the key resources table. Signals were detected using a ChemiDoc Touch MP (Bio-Rad). Images were analyzed using Fiji software (version 2.14.0/1.54f).

#### Cell culture

All cells used in this study, except for MCF10A, were maintained in RPMI 1640 (Nacalai Tesque, cat# 30264-56) supplemented with 10% fetal bovine serum (Biosera, cat# S1810-500, lot# 017BS238), and penicillin-streptomycin (Nacalai Tesque, cat# 26253-84). Cells were cultured at 37°C in a 5% CO_2_ incubator. MCF10A cells were maintained in MEBM basal medium supplemented with BPE, hEGF, insulin, hydrocortisone (MEGM Bullet kit; Lonza, cat# CC-3150), penicillin-streptomycin, and 100 ng/mL cholera toxin (Sigma, C8052). For supplementation assays, a custom BCAA-free RPMI 1640 medium was prepared (Kohjin Bio) and supplemented with 10% dialyzed fetal bovine serum (HyClone, cat# SH30079.03, lot# AB10190992, or Serana, cat# S-FBS-NL-065, lot# 29061022FBS). For depletion culture assays, the BCAA-free media were supplemented with a combination of leucine (Sigma, cat# L8000), isoleucine (Sigma, cat# I2752), valine (Sigma, cat# V0500), sodium KIC (Sigma, cat# K0629), sodium KMV (Sigma, cat# 198978), or sodium KIV (Sigma, cat# 198994). For Human Plasma-like Medium, 10% dialyzed fetal bovine serum and 2-hydroxybutyric acid were added as described previously.^25^ For BCAT1 inhibition experiments, Gabapentin (TCI, cat# G0318) was used to inhibit BCAT1 transaminase activity. GABA (Sigma, cat# A2129) was used as a negative control.

#### Sphere formation assay

MCF7, HCC1143, MDA-MB-231, and Hs578 T were trypsinized and seeded in RPMI 1640 with 10% FBS (Biosera), penicillin/streptomycin, and 0.5% methylcellulose (R&D Systems, cat# HSC011) at 500 cells per well in a 24-well low-binding plate (Sumitomo, cat# MS-90240). 7 days after seeding, cell clusters larger than 40 μm in diameter were counted as spheres. Sphere-forming ability was expressed as sphere-forming efficiency, calculated as the number of spheres divided by the number of seeded cells. For the sphere forming assay of sorted tumor cells, cells were seeded at 1,000 cells per well. At 7–10 days after seeding, the spheres were counted, and the sphere forming efficiency was calculated.

#### Virus production and infection

293 T cells were seeded in DMEM (Nacalai Tesque, cat# 08458-16) supplemented with 10% US Cosmic Calf Serum (HyClone, cat# SH30087.03, lot# AF29528192) and penicillin-streptomycin one day before transfection. For lentivirus production, constructs for expression, shRNA, or CRISPR, together with VSV-G, Rev, and MDLg/pRRE, were co-transfected using PEI Max (MW 40 K; Polysciences, cat# 24765). The next day, culture medium was replaced with fresh medium, and the culture supernatants were collected on subsequent days. Combined supernatants were filtered through a 0.45 μm PES filter. For infection, the lentivirus-containing supernatant was added to the target cells in the presence of 8 μg/mL polybrene. For puromycin selection, the culture medium was replaced with puromycin-containing medium (1–2 μg/mL; Gibco, cat# A1113803) the day after infection. For G418 selection, culture medium containing 800 μg/mL G418 (Nacalai Tesque, cat# 09380-44) was used. Cells were cultured for several days until non-infected cells were eliminated.

#### Generation of stable OLIVe-expressing cells

Cells with stable OLIVe FRET biosensor expression were generated by co-transfecting the OLIVe plasmid (pT2ADW/OLIVe) and the Tol2 transposase expression plasmid (pCAGGS-T2TP). Transfection was performed using X-tremeGENE9 reagent (Roche, cat# 6365779001) per the manufacturer’s instructions. MDA-MB-231 cells with OLIVe were isolated by single cell sorting on a Cell Sorter MA900 (Sony), and several clones were established and confirmed to respond similarly across varying BCAA concentrations in culture.

#### Xenograft transplantation and FRET-based cell sorting

MDA-MB-231 cells with stable OLIVe expression were suspended in transplantation buffer (50% PBS(−) and 50% Matrigel (Corning, cat# 354234) or MatriMix(511) (Nippi, cat# 899001)) and orthotopically transplanted into the 4th left mammary fat pad. For BCAT1 knockdown, MDA-MB-231 cells stably transduced with Tet-on shCtrl or shBCAT1_#2 were used for transplantation. For at least one week after transplantation, doxycycline (10 mg/mL in water) was administered daily by oral gavage at 0.2 mL per mouse.^67^ Thereafter, mice were maintained on an AIN-93G diet supplemented with 0.03% (w/w) doxycycline. Tumor size was measured with a digital caliper and calculated as follows: (long diameter) × (short diameter)^2^ × 1/2 mm^3^. Once a primary tumor formed, it was dissected and subjected to collagenase (Sigma, cat# C9407) digestion supplemented with DNase I (Sigma, cat# DN25). Alternatively, the Tumor Dissociation Kit, human (Miltenyi Biotec, cat# 130-095-929) was used on a gentleMACS Octo Dissociator with Heaters (Miltenyi Biotec) with the 37C_h_TDK_3 program. Red blood cells were removed with RBC lysis buffer. Before cell sorting, dead cells were removed by using the Dead Cell Removal Kit (Miltenyi Biotec, cat# 130-090-101). OLIVe signals were detected with a FACSAriaIII (BD Biosciences) using the following channels: 405 nm laser and 470/40 BP filter for CFP, and 405 nm laser and 510/50 BP filter for YFP-FRET. The FRET ratio was calculated as YFP-FRET signal intensity divided by CFP signal intensity using FACSDiva software, version 9.4 (BD Biosciences). Cells were sorted based on the OLIVe FRET ratio: the top and bottom 10% cells were separated as FRET-High and -Low cells, respectively. Sorted cells were reanalyzed by flow cytometry to confirm successful separation of FRET-High and -Low cells. For cell preparation for *in vivo* limiting dilution assay, cells were counted and resuspended in transplantation buffer. The cell suspension was used for secondary transplantation.

#### Fluorescence microscopy and OLIVe FRET ratio analysis

All microscope images were acquired on a Zeiss AxioObserver7 with Definite Focus 3 using a Plan-Apochromat 20×/0.8 M27 objective lens. CFP and YFP-FRET signals were detected with BP480/40 and BP535/30, respectively, by excitation at BP436/20 and 430 nm using the Colibri 7 LED illumination system with an Axiocam 705 mono camera. Zen software (Zeiss) was used to acquire images. Images were analyzed with Fiji software (version 2.14.0/1.54f). For the BCAA or BCKA response experiments, MDA-MB-231 cells with stable OLIVe expression were maintained in Kohjin Bio’s RPMI 1640-based media containing all amino acids before starting time-lapse imaging at 5 min intervals. After 30 min, the media was removed, and cells were washed with RPMI 1640-based medium without BCAAs to establish a BCAA-free culture condition. 90 min after the media change, BCAAs or BCKAs were added at the following final concentrations: 381 μM for Leu and Ile, and 171 μM for Val (BCAAs); 381 μM for KIC and KMV, and 171 μM for KIV (BCKAs).

For OLIVe FRET analysis, CFP and YFP-FRET images of OLIVe were acquired as described above. Noises were removed using the rolling ball background subtraction plugin in Fiji software. When necessary, cell segmentation was performed with the Cellpose 2.0 algorithm, or regions were manually drawn to define cells for analysis. CFP and YFP-FRET signals were obtained using cell region ROIs. The FRET ratio was calculated as the YFP-FRET signal intensity divided by the CFP signal intensity. For automated FRET ratio analysis, CFP and YFP-FRET images of OLIVe were acquired as described above. Noises were removed using both a median filter and a top-hat transform. Cell segmentation was performed using the Cellpose 2.0 algorithm.^62^ Cell tracking was carried out using the two nearest images at each sampling time point. The cell centroid was calculated in both images based on the previous time point image, and the nearest centroid in the next time point image was identified as the location of the tracked cell. Signal intensities of CFP and YFP-FRET for the tracked cell were measured, and the FRET ratio was calculated as the YFP-FRET signal intensity divided by the CFP signal intensity.

#### Intravital two-photon microscopy

MDA-MB-231 cells with stable OLIVe expression were used for tumor formation in NSG mice, as described above. For imaging, a tumor was exposed via the skin-flap method under isoflurane anesthesia.^68^ YFP-FRET and CFP signals were detected with a two-photon excitation microscope (FV1200MPE-IX83 TPEM5 Invert, Olympus) using a UPLSAPO 20×/0.75 objective lens. The excitation wavelength for both YFP-FRET and CFP was 840 nm delivered by an InSight DeepSee Ultrafast laser (Spectra Physics). For fluorescence imaging, the following emission band-pass filters were used: 460–500 nm for CFP and 520–560 nm for YFP-FRET. The microscope was equipped with two-channel GaAsP detector units. z stack slices were acquired at 10 μm intervals using FluoView application (Olympus), followed by Fiji software. The FRET ratio was displayed as pseudo color using an intensity-modulated display mode.

#### Cell proliferation, cell death, and reactive oxygen species detection assays

Cells were seeded in RPMI 1640 normal growth medium or BCAA-depleted medium as indicated in the figure legend. For the cell proliferation assay, cells were seeded in a 24-well low-binding plate. After 3–6 days of culture, cells were collected and an equal volume of CellTiter-Glo 2.0 cell proliferation assay reagent (Promega, cat# G9243) was added to the cell culture. Samples were transferred to a 96 well white plate, and the luminescent signal was measured using a BioTek Synergy LX plate reader (Agilent). For the cell death assay, cells were seeded in a 24-well low-binding plate. After 3–6 days of culture, cells were collected and washed with PBS(−). They were stained with Alexa Fluor 488-conjugated Annexin V (Thermo, cat# A13201) or APC-conjugated Annexin V (Biolegend, cat# 640941) in staining buffer at room temperature for 15 min. APC-Annexin V positive cells were analyzed on a NovoCyte Quanteon flow cytometer (Agilent). For reactive oxygen species detection, cells were seeded in a 24 well plate. After 3–6 days of culture, CellROX Deep Red Reagent (Thermo, cat# C10422) was added at a final concentration of 200 μM, and the cells were incubated for 30 min to stain. Cells were collected and CellROX Deep Red fluorescence was analyzed on a NovoCyte Quanteon flow cytometer (Agilent). *tert*-Butyl hydroperoxide (TBHP) was used at 200 μM as a positive control to induce reactive oxygen species.

#### Stem cell marker assays

For CD24/CD44 analysis, tumor cells were stained on ice for 30 min with APC-conjugated anti-CD44 antibody (Biolegend, cat# 103012, clone IM7) and APC/Cy7-conjugated anti-CD24 antibody (Biolegend, cat# 311132, clone ML5). Propidium iodide at 0.5 μg/mL was used for dead cell staining. Stained cell samples were analyzed on a NovoCyte Quanteon flow cytometer (Agilent). Acquired FCS files were analyzed with FlowJo software (version 10.10.0) to calculate the OLIVe FRET ratio with CD44 and CD24 signals. For ALDH activity, the recently developed ALDH activity probe C5S-A was used.^49^ Cells were seeded onto a collagen-coated glass-bottom dish and stained for 30 min with C5S-A at a final concentration of 1 μM. The fluorescent signal of C5S-A was detected using a BP690/50 filter with excitation at BP640/30 and a 630 nm LED of the Colibri 7 LED illumination system. OLIVe FRET signals were detected as described in the “Fluorescence microscopy” section. Signal intensities for each channel were analyzed with Fiji application.

#### Cell competition assay

Tumors formed by OLIVe-expressing MDA-MB-231 cells were dissected from recipients, and tumor cells were sorted based on the OLIVe FRET ratio as described above. FRET-High and -Low cells were stained with CellTrace Far Red (Thermo, cat# C34572) and CellTrace Yellow (Thermo, cat# C34573), respectively, following the manufacturer’s protocol. The stained cells were mixed at a 1:1 ratio and seeded for culture. After 3 or 6 days, the cells were stained and analyzed on a NovoCyte Quanteon flow cytometer (Agilent).

#### Amino acid uptake assay

Cells were seeded in a 96-well glass-bottom plate (EZVIEW culture plate LB, Iwaki, cat# 5866-096). The Amino Acid Uptake Assay Kit (Dojindo, cat# UP04/346-09891) was used, and the assay was carried out following the manufacturer’s instructions. Briefly, cells were washed with HBSS, and the amino acid analog BPA was added for cellular uptake. After a 5-min incubation, excess BPA was washed out with HBSS, and the BPA probe was added. Fluorescence from the probe-BPA complex was captured with an AxioObserver7 microscope using a DAPI filter set and analyzed with Fiji software.

#### Amino acid quantification

Peripheral blood samples were collected from the eye vein of mice bearing a breast tumor using a heparin-coated capillary. Plasma was separated by centrifugation, and metabolites were extracted with methanol/acetonitrile (1:1, v/v). For cultured cell samples, metabolites were extracted with 80% methanol. After removal of insoluble materials by centrifugation, the supernatant equivalent to 2 μL of peripheral blood plasma or 1 × 10^5^ cells was collected and vacuum-dried using a SpeedVac. Samples were reacted with NBD-F, and amino acids were quantified as previously described^11^ using an HPLC-fluorescence detection system (Shimadzu).

#### NMR-based metabolic analysis

To monitor BCAA-BCKA conversion, cells were seeded in normal growth media one day before labeling. The next day, the cell culture medium was replaced with labeling medium containing either 170 μM [(U)-^13^C, ^15^N]-Val (Cambridge Isotope Laboratories) and 30 μM unlabeled KIV, or 170 μM unlabeled Val and 30 μM [(U)-^13^C]-KIV (Cambridge Isotope Laboratories), and cells were cultured for 30 min. After two washes with cold PBS(−), metabolites were extracted with 80% methanol on ice for 10 min. After removal of insoluble by centrifugation, supernatants were vacuum-dried using a SpeedVac. ^1^H-^13^C heteronuclear single-bond correlation (HSQC) analysis was performed as previously described.^11^

For real-time metabolic analysis by operando NMR, we used [(U)-^13^C]-KIV in a protocol essentially as described previously.^33^ In brief, 3 × 10^6^ cells were resuspended in IMDM with 10% dialyzed FBS, loaded into an NMR rotor, and [(U)-^13^C]-KIV solution was added just before measurement to a final concentration of 170 μM. HSQC spectra were collected on an 800 MHz Avance III 800US Plus NMR (Bruker). The spin rate was 3,200 Hz at the magic angle, and the temperature was set to 300 K (27°C). Spectra were analyzed with MestReNova software (version 15.0.0) and presented for a total of 18 cycles with 64 scans per cycle.

#### Cancer Cell Line Encyclopedia (CCLE) dataset analysis

Expression datasets for BCAT1 and BCAT2 in breast cancer cell lines from CCLE were retrieved from the DepMap portal developed by the Broad Institute (version 22Q4).^26–28^ Subtype classification is based on previous reports.^40,69,70^ CCLE gene expression data in breast cancer cell lines were extracted according to cell line classification, following the report by Dai et al.^69^ Cell lines used in the analysis were selected based on the reports by Neve et al. and Prat et al.^40,70^ In particular, the claudin-low subtype was defined as in the report by Prat et al.^40^ BCAT expression was shown on a log2 scale. Subject numbers for each subtype are as follows: luminal A (*n* = 9), luminal B (*n* = 4), HER2-positive (*n* = 7), basal-like (*n* = 13), and claudin-low (*n* = 6).

#### Breast cancer patient dataset analysis

The gene expression and clinical data used in this study were retrieved from public databases, TCGA-BRCA (The Cancer Genome Atlas - Breast Cancer cohort)^36^ and METABRIC (Molecular Taxonomy of Breast Cancer International Consortium),^34,35^ downloaded from the Genomic Data Commons (GDC) portal (https://portal.gdc.cancer.gov/) and cBioPortal (https://www.cbioportal.org/), respectively. For data preprocessing, R (version 4.2.1) was used. Clinical data from Curtis et al.^34^ and Pereira et al.^35^ were combined for the METABRIC dataset, including PAM50 classification and other patient details. Gene expression data from Illumina microarrays were used. Only patients with complete expression and clinical data were included for analysis, yielding 1,082 and 1,980 samples from TCGA-BRCA and METABRIC, respectively.

To classify samples into claudin-low and non-claudin-low subtypes, the ClaudinLow R package (https://github.com/clfougner/ClaudinLow) was used. Gene expression data were preprocessed to meet its requirements by removing duplicate samples and entries missing Entrez Gene IDs. A list of claudin-low signature genes was obtained from the genefu package, and gene expression data were compared to identify common genes. Expression values of these genes were standardized using the scale function to have a mean of 0 and a standard deviation of 1. Classification of the samples was performed using the claudin-low function with Euclidean distance-based clustering, and results were integrated with clinical data to analyze claudin-low tumor characteristics. To validate the claudin-low classification, further molecular subtyping was performed. PAM50 classification, derived from TCGA clinical data, was used to categorize breast cancer subtypes (luminal A, luminal B, HER2-positive, basal-like, normal-like). The PAM50 gene signature was matched with the gene expression data, and the classification results were integrated with clinical data to assess the relationship with claudin-low. Boxplots of gene expression levels were generated using ggplot2, and statistical comparisons between groups were performed using t-tests implemented in the ggpubr package.

#### RNA-seq analysis

For RNA-seq analysis, MDA-MB-231 cells with stable OLIVe expression were transplanted into NSG mice, and cells were isolated after tumor formation, as described in the “Xenograft transplantation and FRET-based cell sorting” section. OLIVe FRET-High and -Low populations, each with more than 10,000 cells, were directly sorted into TRI Reagent (Sigma, cat# T9424). Libraries were prepared using modified Quartz-Seq and Quartz-Seq2 methods.^60,61^ Reverse transcription was performed with the RT primer and SuperScriptIII (Thermo Fisher, cat# 18080-044). Samples were purified using the DNA Clean & Concentrator-5 kit (Zymo Research, cat# D4014), and poly-A tailing was performed using dATPs (Thermo, cat# 10216-018), RNase H (Thermo, cat# 18021-071), and TdT enzyme (Roche, cat# 3333574001). Second-strand synthesis was performed with the tagging primer and MightyAmp DNA Polymerase Ver.2 (Takara, cat# R071A), followed by PCR enrichment. Samples were first purified with the DNA Clean & Concentrator-5 kit, then with Agencourt AMPure XP (Beckman Coulter, cat# A63880). DNA was fragmented using a Covaris S220 ultrasonicator under the following conditions: duty factor 10%, peak incident 175, cycles/burst 100, time 600 s, and then purified with the DNA Clean & Concentrator-5 kit. DNA fragmentation was verified using the BioAnalyzer High sensitivity DNA kit (Agilent, cat# 5067-4626) with a 2100 Bioanalyzer (Agilent). End repair and dA-tailing were conducted with the KAPA Hyper Prep Kit (KAPA Biosystems, cat# KK8502), followed by adaptor ligation with the SeqCap Adapter Kit A (Roche, cat# 07141530001). Adaptor dimers were removed by purification with Agencourt AMPure XP. DNA was enriched with the KAPA HiFi HotStart ReadyMix (included in the KAPA Hyper Prep Kit), followed by two rounds of purification with Agencourt AMPure XP. Library DNA was quantified using a qPCR-based method with the Collibri Library Quantification Kit (Thermo, cat# A38524100). Sequencing was performed on an Illumina NextSeq 500 instrument using the NextSeq 500 High-Output v2.5 Kit, 75 Cycles (Illumina, cat# 20024906). Raw FASTQ files were trimmed to remove poly-G artifacts using Trim Galore (v0.6.11). Trimmed reads were mapped to the human genome (GRCh38) using HISAT2 (v2.2.1). The resulting SAM files were converted to BAM, filtered for primary alignments, sorted, and indexed using SAMtools (v1.21). Read counts for each gene were obtained using featureCounts (v2.0.3). Differentially expressed gene (DEG) analysis and Gene Set Enrichment Analysis using the Molecular Signatures Database (MSigDB)^71–73^ were performed with RNAseqChef (v1.1.6).^63^

### QUANTIFICATION AND STATISTICAL ANALYSIS

Statistical analyses were performed using GraphPad Prism version 10.3.1 (GraphPad Software Inc.). Data are presented as mean ± SD or SEM., as indicated in the figure legends. Statistical significance was assessed using simple linear regression analysis, unpaired or paired two-tailed Student’s *t* test (with Welch’s correction where appropriate), two-tailed Mann-Whitney U test, one-way ANOVA, Welch’s ANOVA, two-way ANOVA, or Gehan-Breslow-Wilcoxon test. Post hoc multiple-comparison corrections were applied when appropriate, and the statistical tests used are indicated in each figure legend. For the distribution analysis of two-photon microscopy imaging of the OLIVe FRET ratio in a tumor formed *in vivo*, statistical variance was evaluated by *F* test, and the absolute residual value was used to visualize variance in FRET ratios. Statistical significance is indicated by asterisks based on *p* values: * <0.05, ** <0.01, *** <0.005, and **** <0.001. n.s., not significant.

## Supplementary Material

1

SUPPLEMENTAL INFORMATION

Supplemental information can be found online at https://doi.org/10.1016/j.celrep.2026.117630.

## Figures and Tables

**KEY RESOURCES TABLE:** 

REAGENT or RESOURCE	SOURCE	IDENTIFIER

Antibodies		

Mouse monoclonal anti-BCAT1 (clone 51/ECA39)	BD Biosciences	Cat# 611271; RRID: AB_398799
Rabbit monoclonal anti-BCAT2 (clone D8K3O)	Cell Signaling Technology	Cat# 79764; RRID: AB_2799939
Rabbit monoclonal anti-BCKDHA (clone E4T3D)	Cell Signaling Technology	Cat# 90198; RRID: AB_2800155
Rabbit monoclonal anti-pS293-BCKDHA (clone E2V6 B)	Cell Signaling Technology	Cat# 40368; RRID: AB_2799176
Rabbit polyclonal anti-BCKDHB	Abcam	Cat# ab201225; RRID: AB_2858261
Mouse monoclonal anti-BCKDK (clone E–12)	Santa Cruz Biotechnology	Cat# sc-374425; RRID: AB_10988235
Rabbit monoclonal anti-S6K (clone 49D7)	Cell Signaling Technology	Cat# 2708; RRID: AB_390722
Rabbit monoclonal anti-pT389-S6K (clone 108D2)	Cell Signaling Technology	Cat# 9234; RRID: AB_2269803
Rabbit monoclonal anti-4E-BP1 (clone 53H11)	Cell Signaling Technology	Cat# 9644; RRID: AB_2097841
Rabbit monoclonal anti-pT37/46–4 E-BP1 (clone 236B4)	Cell Signaling Technology	Cat# 2855; RRID: AB_560835
Rat monoclonal anti-MSI1 (clone 14H1)	Thermo Fisher Scientific	Cat# 14-9896-82; RRID: AB_10598804
Rabbit monoclonal anti-MSI2 (clone EP1305Y)	Abcam	Cat# ab76148; RRID: AB_1523981
Rabbit monoclonal anti-c-MYC (clone D84C12)	Cell Signaling Technology	Cat# 5605; RRID: AB_1903938
Rabbit monoclonal antiHistone H3K9me3 (clone D4W1U)	Cell Signaling Technology	Cat# 13969; RRID: AB_2798355
Mouse monoclonal antiHistone H3 (clone 1G1)	Santa Cruz Biotechnology	Cat# sc-517576; RRID: AB_2848194
Mouse monoclonal anti-V5-tag (clone 7/4)	BioLegend	Part# 77620 (in cat# 699903); RRID: AB_2566386
Mouse monoclonal anti-T7-tag	Novagen	Cat# 69522; RRID: AB_11211744
Mouse monoclonal anti-β-actin (clone 8H10D10)	Cell Signaling Technology	Cat# 3700; RRID: AB_2242334
Rabbit monoclonal anti-β-actin (clone 13E5)	Cell Signaling Technology	Cat# 4970; RRID: AB_2223172
Mouse monoclonal anti-HSP90 (clone F-8)	Santa Cruz Biotechnology	Cat# sc-13119; RRID: AB_675659
APC-conjugated anti-CD44 antibody, clone IM7	BioLegend	Cat# 103012; RRID: AB_312962
APC/Cy7-conjugated anti-CD24 antibody, clone ML5	BioLegend	Cat# 311132; RRID: AB_2566347

Chemicals, peptides, and recombinant proteins		

Alexa Fluor 488-conjugated Annexin V	Thermo Fisher Scientific	Cat# A13201
APC-conjugated Annexin V	BioLegend	Cat# 640941
Matrigel Basement Membrane Matrix	Corning	Cat# 354234
MatriMix(511)	Nippi	Cat# 899001
Collagenase from Clostridium histolyticum	Sigma-Aldrich	Cat# C9407
DNase I	Sigma-Aldrich	Cat# DN25
BCAA-free custom RPMI 1640 medium	Kohjin Bio	N/A (custom order)
Fetal bovine serum (FBS)	Biosera	Cat# S1810-500, lot# 017BS238
Dialyzed fetal bovine serum	HyClone	Cat# SH30079.03, lot# AB10190992
Dialyzed fetal bovine serum	Serana	Cat# S-FBS-NL-065, lot# 29061022FBS
US Cosmic Calf Serum	HyClone	Cat# SH30087.03, lot# AF29528192
L-Leucine	Sigma-Aldrich	Cat# L8000
L-Isoleucine	Sigma-Aldrich	Cat# I2752
L-Valine	Sigma-Aldrich	Cat# V0500
Sodium α-ketoisocaproate (KIC; sodium 4-methyl-2-oxopentanoate)	Sigma-Aldrich	Cat# K0629
Sodium α-keto-β-methylvalerate (KMV)	Sigma-Aldrich	Cat# 198978
Sodium α-ketoisovalerate (KIV)	Sigma-Aldrich	Cat# 198994
Gabapentin (BCAT1 chemical inhibitor)	TCI	Cat# G0318
GABA (γ-aminobutyric acid; negative control)	Sigma-Aldrich	Cat# A2129
Methylcellulose	R&D Systems	Cat# HSC011
PEI Max, mw 40 K (Polyethylenimine)	Polysciences	Cat# 24765
X-tremeGENE 9 DNA Transfection Reagent	Roche/Thermo Fisher Scientific	Cat# 6365787001
MightyAmp DNA Polymerase Ver.2	Takara Bio	Cat# R071A
SuperScript III Reverse Transcriptase	Thermo Fisher Scientific	Cat# 18080-044
dATPs	Thermo Fisher Scientific	Cat# 10216-018
RNase H	Thermo Fisher Scientific	Cat# 18021-071
TdT enzyme (Terminal deoxynucleotidyl transferase)	Roche	Cat# 3333574001
Agencourt AMPure XP beads	Beckman Coulter	Cat# A63880
KAPA Hyper Prep Kit	KAPA Biosystems (Roche)	Cat# KK8502
SeqCap Adapter Kit A	Roche	Cat# 07141530001
KAPA HiFi HotStart ReadyMix	KAPA Biosystems (Roche)	Included in Cat# KK8502
Collibri Library Quantification Kit	Thermo Fisher Scientific	Cat# A38524100
DNA Clean & Concentrator-5 Kit	Zymo Research	Cat# D4014
BioAnalyzer High Sensitivity DNA Kit	Agilent	Cat# 5067-4626
CellTiter-Glo 2.0 Cell Viability Assay reagent	Promega	Cat# G9243
CellROX Deep Red Reagent	Thermo Fisher Scientific	Cat# C10422
CellTrace Far Red Cell Proliferation Kit	Thermo Fisher Scientific	Cat# C34572
CellTrace Yellow Cell Proliferation Kit	Thermo Fisher Scientific	Cat# C34573
[(U)-^13^ C,^15^N]-valine	Cambridge Isotope Laboratories	CNLM-442-H-0.25
[(U)-^13^C]- Na-KIV	Cambridge Isotope Laboratories	CLM-4418-0.25
Dimethyl sulfoxide (DMSO)	Nacalai Tesque	13408-64
C5S-A dye (ALDH activity assay, red-channel)	Oe et al.^49^	N/A
Amino Acid Uptake Assay Kit (BPA probe)	Dojindo	Cat# UP04/346-09891
MycoAlert Mycoplasma Detection Kit	Lonza	Cat# LT07-418

Critical commercial assays		

Tumor Dissociation Kit, human	Miltenyi Biotec	Cat# 130-095-929
Dead Cell Removal Kit	Miltenyi Biotec	Cat# 130-090-101

Deposited data		

CCLE gene expression dataset (version 22Q4)	DepMap Portal (Broad Institute)	https://depmap.org/portal/
TCGA-BRCA gene expression and clinical data	Genomic Data Commons (GDC) Portal	https://portal.gdc.cancer.gov/
METABRIC gene expression and clinical data	cBioPortal	https://www.cbioportal.org/
NMR spectral data series	This study	Metabolomics Workbench: PR003139 doi: https://doi.org/10.21228/M8XS06
unprocessed RNA-seq reads	This study	DDBJ BioProject: PRJDB42235

Experimental models: Cell lines		

MCF7	RIKEN BRC	RIKEN BRC Cat# RCB1904; CVCL_0031
BT-474	Chiba University (lab stock)	CVCL_0179
MDA-MB-453	RIKEN BRC	RIKEN BRC Cat# RCB1192; CVCL_0418
SK-BR-3	Chiba University (lab stock)	CVCL_0033
HCC1143	Gift from Dr. Noriko Gotoh (Kanazawa University)	CVCL_1245
MDA-MB-231	Kato et al.^58^; lab stock	CVCL_0062
Hs578 T	Chiba University (lab stock)	CVCL_0332
MCF10A	Gift from Dr. Yasuyuki Fujita (Kyoto University)	CVCL_0598
HEK293T	Lab stock	CVCL_0063

Experimental models: Organisms/strains		

NOD.Cg-*Prkdc^scid^ Il2rg^tm1Wjl^*/SzJ mouse, NSG mouse, female, 7 weeks old	Jackson Laboratory	N/A
AIN-93G diet with 0.03% (w/w) Doxycycline	Oriental Bio Service	N/A

Oligonucleotides		

shCtrl (shTurboGFP): CGTGATCTTCACCGACAAGAT	This study	N/A
shBCAT1_#2: GAG CCCAGTGGGACCTTATTT	This study	N/A
shBCAT1_16 + 3: TGCCCAATGTGAAGCAGTAGA	This study	N/A
shBCAT1_Q4: AAGAATGGGTCCCATATTCAA	This study	N/A
shBCAT1_#1: CCCAATGTGAAGCAGTAGATA	Wang et al.^10^	N/A
shBCAT2: GTGGGAACCATGAACATCTTT	This study	N/A
sgCtrl: GCGAGGTATTCGGCTCCGCG	Zurlo et al.^59^	N/A
sgBCKDK_#1: GTCGGCCATCGACGCGGCAG	This study	N/A
sgBCKDK_#2: CTACCTTACCACGTGCAGTA	This study	N/A
sgBCKDK_#3: CATCGACGCGGCAGCGGAGA	This study	N/A
sgBCAT1_#1: TAGTGCAAAGCTGATGAGCC	This study	N/A
sgBCAT1_#2: AGACTAGCAGATGTTGAATA	This study	N/A
sgBCAT1_#3: TTTTCCTTTAAAATGGTAGC	This study	N/A
sgBCAT2_#4: TCGGCAACTACAAGTTAGGT	This study	N/A
sgBCAT2_#5: CCGCACATAGAGGCTGGTGC	This study	N/A
sgc-MYC_#1: GCCGTATTTCTACTGCGACG	This study	N/A
sgc-MYC_#2: GCCGTCGTTGTCTCCCCGAA	This study	N/A
sgc-MYC_#3: TGCGTAGTTGTGCTGATGTG	This study	N/A
sgc-MYC_#4: AACGTTGAGGGGCATCGTCG	This study	N/A
RT primer: 5′ -TATAGAATTCGCGGCCGCTCGCGATAATACGACTCACTATAGGGCGT_24_-3′	Sasagawa et al.^60,61^	N/A
Tagging primer: 5′ -TATAGAATTCGCGGCCGCTCGCGAT_24_-3′	Sasagawa et al.^60,61^	N/A
PCR primer (5′NH2-C6 amination): 5’-(NH2)-GTATAGAATTCGCGGCCGCTCGCGAT-3′	Sasagawa et al.^60,61^	N/A

Software and algorithms		

FACSDiva, version 9.4	BD Biosciences	N/A
FlowJo, version 10.10.0	BD Biosciences	N/A
MestReNova, version 15.0.0	Mestrelab Research	N/A
Zen, version 3.4.91.00000	Carl Zeiss	N/A
Fiji (ImageJ), version 2.14.0/1.54f	NIH	N/A
Cellpose 2.0	Pachitariu and Stringer^62^	N/A
RNAseqChef, version 1.1.6	Etoh and Nakao^63^	N/A
GraphPad Prism, version 10.3.1	GraphPad Software Inc.	N/A

## Highlights

- Claudin-low triple-negative breast cancer relies on branched-chain amino acids
- Cancer stem cell potential is sustained by elevated intracellular branched-chain amino acids
- BCAT1 enzyme mediates branched-chain amino acid production and drives tumor growth
- The BCAT1-BCKDHc axis offers a therapeutic target in triple-negative breast cancer
