# Supplementary material for: Distinct metabolic dependency on BCAA defines cancer stemness and malignancy in human triple-negative breast cancer
Source: Cell Rep. Author manuscript; Available in PMC 2026 Aug 16. (PMC13477800; doi:10.1016/j.celrep.2026.117630)
Supplement: Supplementary file 1 [file NIHMS2199960-supplement-1.pdf]

Cell Reports, Volume *45*

Supplemental information

Distinct metabolic dependency on

BCAA defines cancer stemness and malignancy

in human triple-negative breast cancer

Kenkyo Matsuura, Ririko Shinonaga, Mizuki Yamamoto, Yoshiki Yamamoto, Hsin Chen, Ayaka Maeno, Kayo Okuda, Haruki Inoue, Sota Imotani, John Glushka, Koji Miki, Yukako Watanabe, Mamoru Takada, Hironori Kaji, Jun-ichiro Inoue, Ayuna Hattori, Hiromi Imamura, and Takahiro Ito

Figure S1

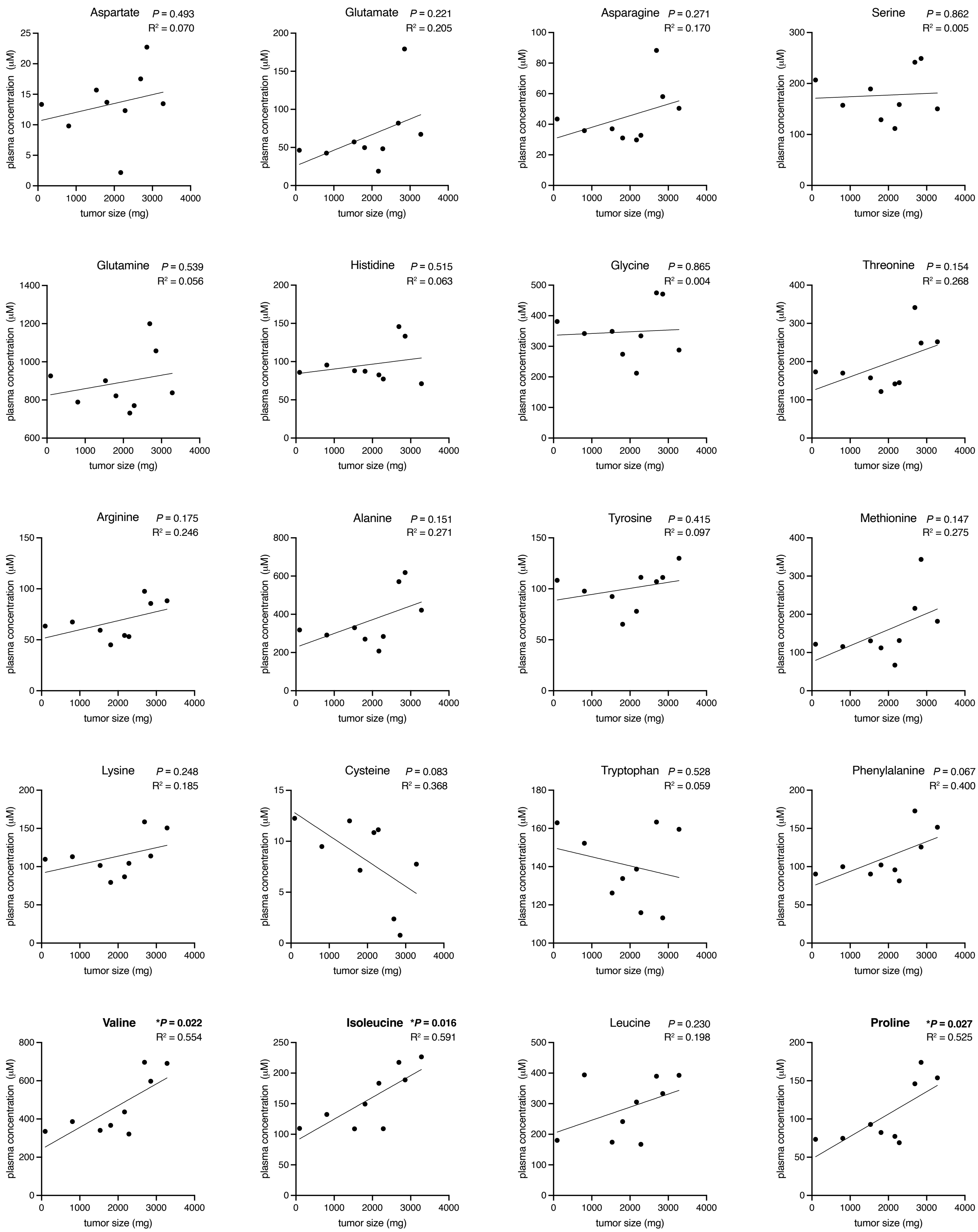

Figure S1: Plasma amino acid levels in tumor-bearing mice.

Plasma amino acid concentrations in mice bearing tumors were analyzed as described in the METHOD DETAILS section. n = 9 for each amino acid. Related to Figure 1A. Pearson's correlation coefficient (R^2) and *P* value are reported.

Statistical significance was indicated by an asterisk based on *P* values: * <0.05

Figure S2

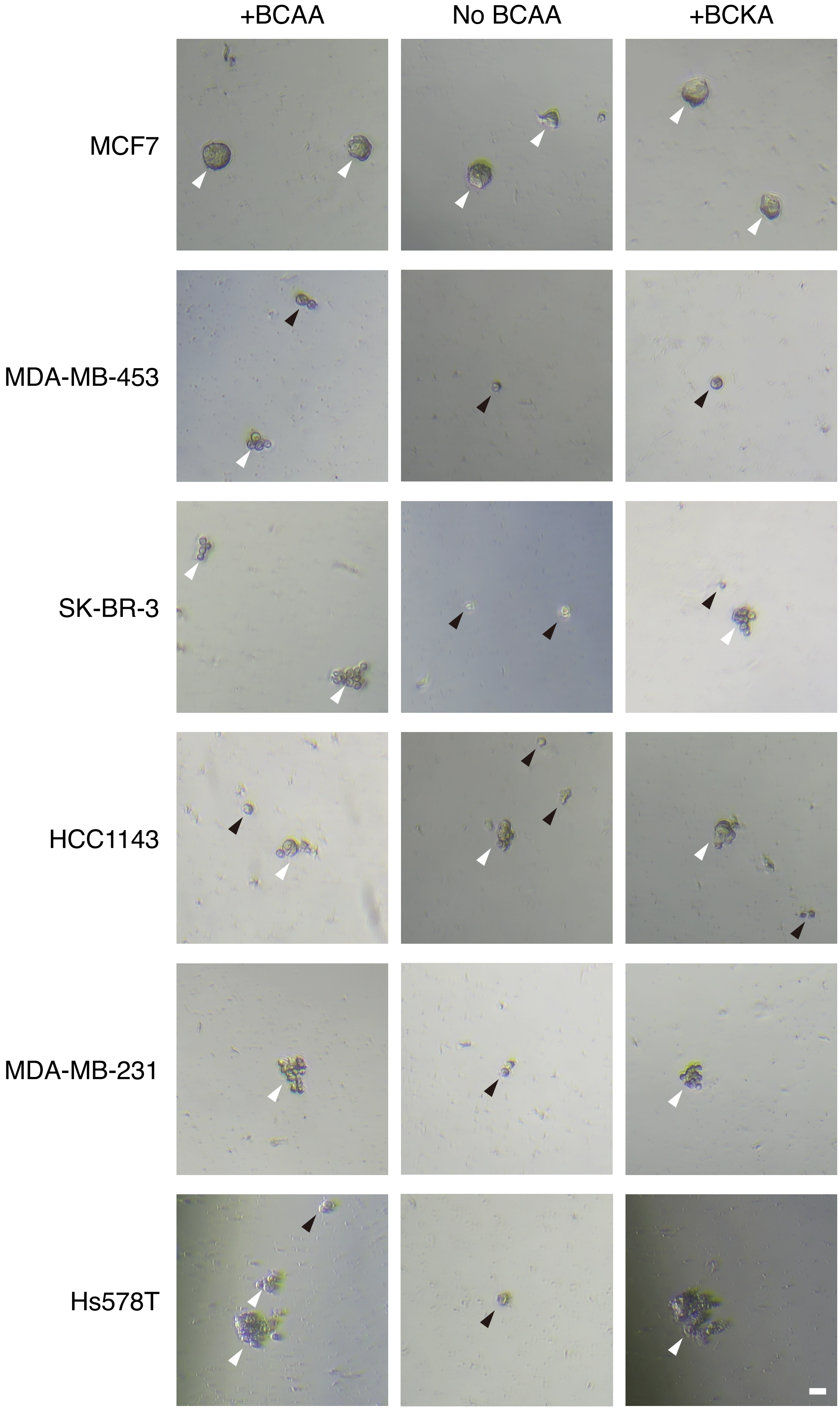

Figure S2: Representative images of spheres.

Sphere formation assays were performed on six breast cancer cell lines as described in the METHOD DETAILS section. After sphere formation, representative phase-contrast images were acquired on a Zeiss Primovert with a Plan-Achromat 4x/0.10 phase contrast lens. Spheres with a diameter of >40 μm were counted (white arrowheads). Clusters with a diameter <40 μm (black arrowheads) were not counted as spheres. The scale bar indicates 40 μm. Related to Figure 1B.

Figure S3

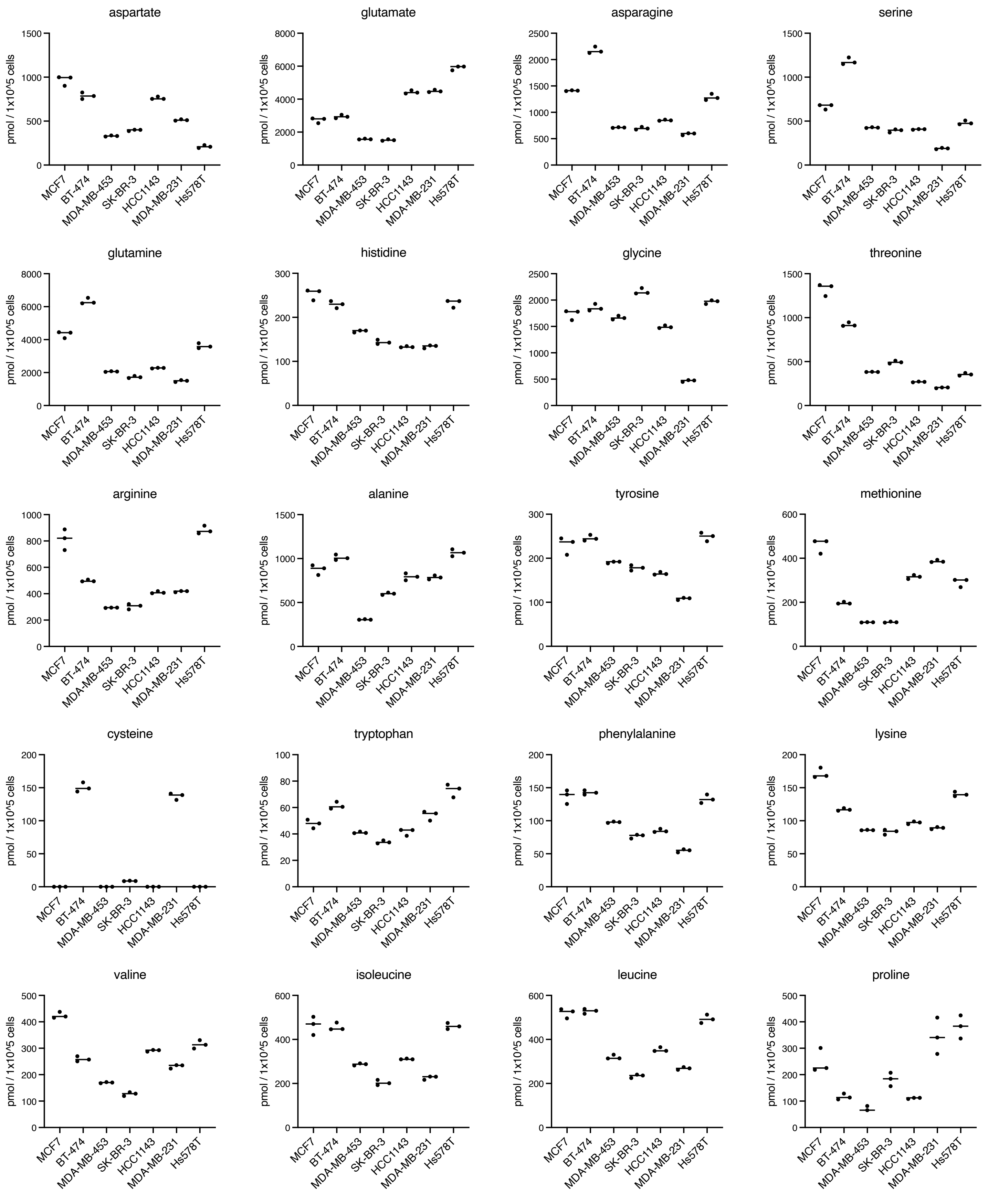

Figure S3: Intracellular amino acid concentrations of human breast cancer cell lines.

Amino acids were quantified as described in the METHOD DETAILS section. Luminal subtype MCF7 and BT-474 cells, HER2-positive subtype MDA-MB-453 and SK-BR-3 cells, basal-like TNBC subtype HCC1143 cells, and claudin-low TNBC subtype MDA-MB-231 and Hs578T cells were analyzed. n = 3 per cell line. Data are presented with mean value (black bar).

Figure S4

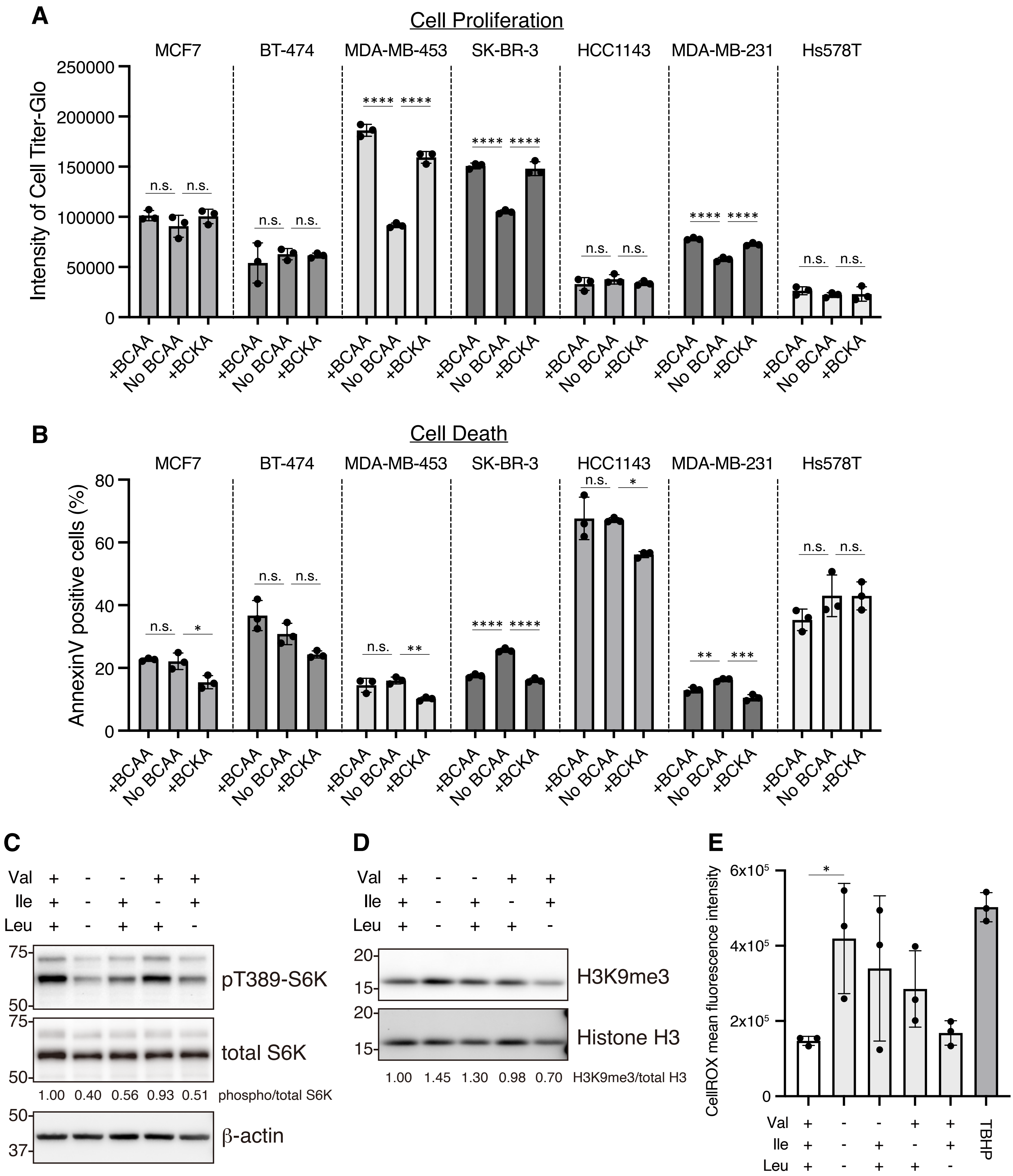
A
Cell Proliferation
MCF7
BT-474
MDA-MB-453
SK-BR-3
HCC1143
MDA-MB-231
Hs578T
Intensity of Cell Titer-Glo
250000
200000
150000
100000
50000
0
n.s.
****
+BCAA
No BCAA
+BCKA
B
Cell Death
AnnexinV positive cells (%)
80
60
40
20
0
n.s.
*
**
***
****
+BCAA
No BCAA
+BCKA
C
Val
Ile
Leu
75
50
37
pT389-S6K
total S6K
1.00 0.40 0.56 0.93 0.51 phospho/total S6K
β-actin
D
20
15
H3K9me3
Histone H3
1.00 1.45 1.30 0.98 0.70 H3K9me3/total H3
E
CellROX mean fluorescence intensity
6x10^5
4x10^5
2x10^5
0
*
Val
Ile
Leu
TBHP

Figure S4: Effects of BCAAs and BCKAs on breast cancer cell lines *in vitro*.

(A) Cell proliferation of breast cancer cell lines in the presence or absence of BCAAs/BCKAs. Each breast cancer cell line was cultured in suspension in the specified growth medium without BCAAs, with BCAAs, or with BCKAs. After 3 days of culture, cells were collected, mixed with an equal volume of CellTiter-Glo2.0 assay reagent, and luminescence was measured. n = 3 per condition. Data are presented as mean ± SD. One-way ANOVA with Tukey's multiple-comparison test was performed. Related to Figure 1B.

(B) Cell survival of breast cancer cell lines cultured in the presence or absence of BCAAs/BCKAs, as described in (A). After 3 days of culture, cells were collected and stained with Alexa Fluor 488-conjugated Annexin V. The Annexin V-positive population was analyzed by flow cytometry. n = 3 per condition. Data are presented as mean ± SD. One-way ANOVA with Tukey's multiple-comparison test was performed. Related to Figure 1B.

(C) mTORC1 activity in medium lacking all or a single BCAA. Phospho-T389 (pT389) S6K and total S6K protein levels were analyzed by immunoblot. β-actin served as a loading control. Band intensity was measured using Fiji software, and the pT389-to-total S6K ratios are shown under each lane. Related to Figure 1C.

(D) Epigenetic modification in medium lacking all or a single BCAA. Histone H3 trimethylation at K9 (H3K9me3) and total histone H3 protein levels were analyzed by immunoblot. The H3K9me3-to-total histone H3 ratios are shown under each lane. Related to Figure 1C.

(E) Reactive oxygen species (ROS) levels in medium lacking all or a single BCAA. Cells were stained with CellROX Deep Red reagent and analyzed by flow cytometry to quantify intracellular ROS. Cells treated with tert-butyl hydroperoxide (TBHP) served as a positive control for ROS induction. n = 3 per condition. Data are presented as mean ± SD. One-way ANOVA with Tukey's multiple-comparison test was performed. Related to Figure 1C.

Statistical significance was indicated by asterisks based on *P* values: * <0.05, ** <0.01, *** <0.005, and **** <0.001. n.s., not significant.

Figure S5

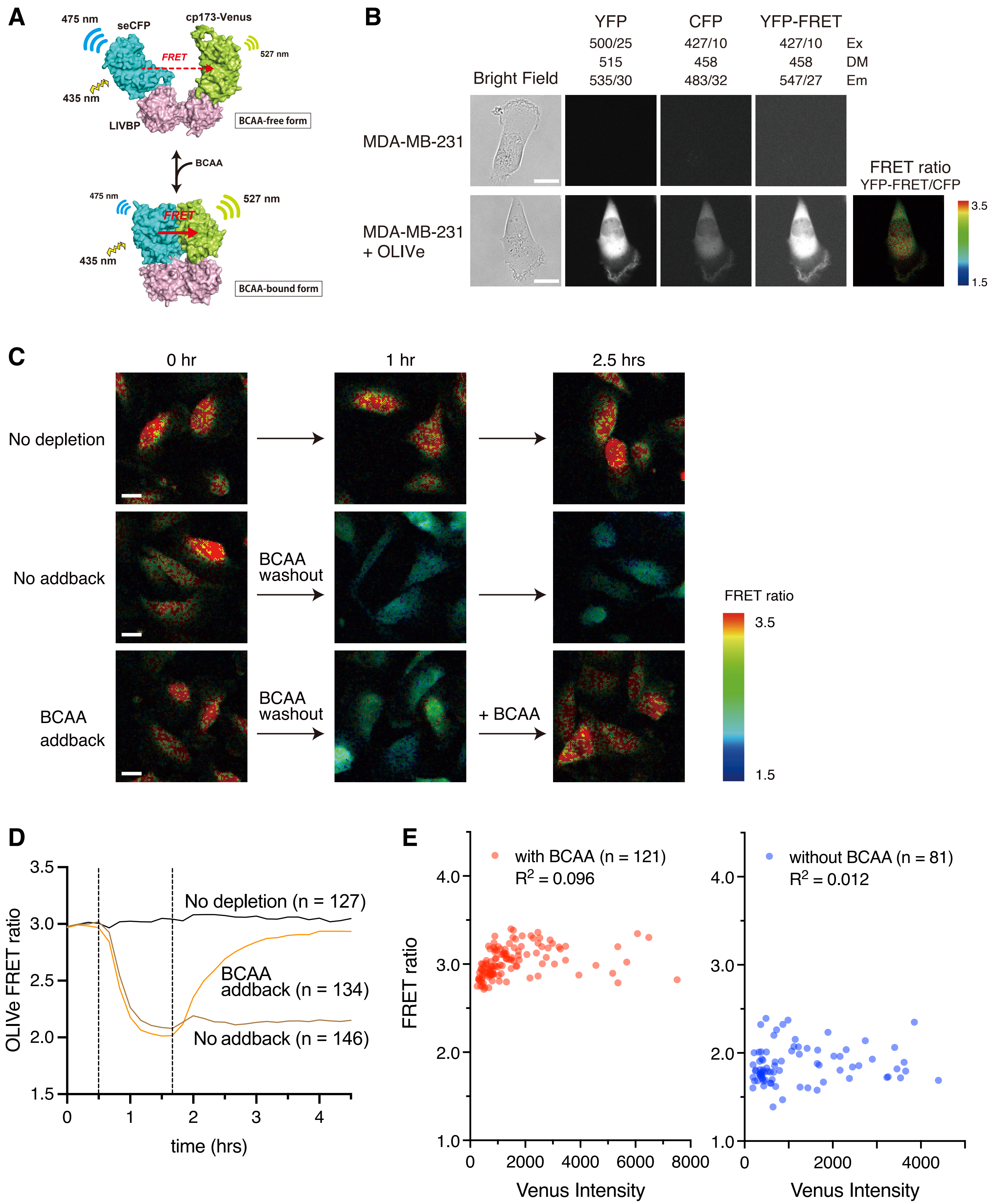

A
475 nm
seCFP
cp173-Venus
FRET
527 nm
435 nm
LIVBP
BCAA-free form
BCAA
475 nm
527 nm
FRET
435 nm
BCAA-bound form
B
YFP
CFP
YFP-FRET
500/25
515
535/30
427/10
458
483/32
427/10
458
547/27
Ex
DM
Em
Bright Field
MDA-MB-231
MDA-MB-231
+ OLIVe
FRET ratio
YFP-FRET/CFP
3.5
1.5
C
0 hr
1 hr
2.5 hrs
No depletion
No addback
BCAA washout
BCAA addback
BCAA washout
+ BCAA
FRET ratio
3.5
1.5
D
OLIVe FRET ratio
No depletion (n = 127)
BCAA addback (n = 134)
No addback (n = 146)
time (hrs)
E
with BCAA (n = 121)
R2 = 0.096
without BCAA (n = 81)
R2 = 0.012
FRET ratio
Venus Intensity
Venus Intensity

Figure S5: Characterization of OLIVe FRET biosensor in breast cancer cells.

(A) Schematic representation of the BCAA biosensor OLIVe. OLIVe consists of seCFP and cp173-Venus (a variant of yellow fluorescent protein, YFP), linked by a BCAA-binding domain from the bacterial LIVBP protein. When BCAAs are present, OLIVe binds them and undergoes a conformational change that triggers FRET upon excitation of seCFP with a 435 nm light source. Consequently, fluorescence at 527 nm is emitted from cp173-Venus. Related to Figures 1D and 1E.

(B) OLIVe signals in MDA-MB-231 cells. Parental and OLIVe-expressing MDA-MB-231 cells were imaged by fluorescence microscopy. The signal acquisition settings are shown at the top. Ex: excitation bandpass filter; DM: dichroic mirror; Em: emission bandpass filter. Numbers indicate wavelength in nm. The FRET ratio was calculated as the YFP-FRET signal divided by the CFP signal and presented as a pseudocolored image (bottom right). Scale bars indicate 20 μm. Related to Figures 1D and 1E.

(C, D) OLIVe response to BCAAs. (C) MDA-MB-231 cells stably expressing OLIVe were cultured in BCAA-free media for 1 hr. Subsequently, cells were replenished with media with or without BCAAs and cultured for another 1.5 hrs. The OLIVe FRET ratio was displayed as pseudocolor. Control cells with no BCAA depletion were cultured in BCAA-containing complete media (No Depletion). Scale bars indicate 20 μm. (D) Time course of the OLIVe FRET signals. Fluorescent signals were acquired at 10-min intervals for 4.5 hrs to calculate FRET ratios. n = 127 for No depletion, n = 134 for BCAA addback, and n = 146 for No addback conditions. Mean values are shown. Related to Figures 1D, 1E, and 4C.

(E) The FRET ratio is independent of OLIVe protein expression level. Fluorescence signals from cp173-Venus, acquired with an excitation filter at 500/25 nm and an emission filter at 535/30 nm, were used as an indicator of OLIVe protein expression level. The graphs show the relationships between the OLIVe FRET ratios and Venus fluorescence intensity in medium with or without BCAAs. n = 121 and n = 81 for with BCAA and without BCAA conditions, respectively. Pearson's correlation coefficients (R^2) are shown. Related to Figures 1D and 1E.

Figure S6

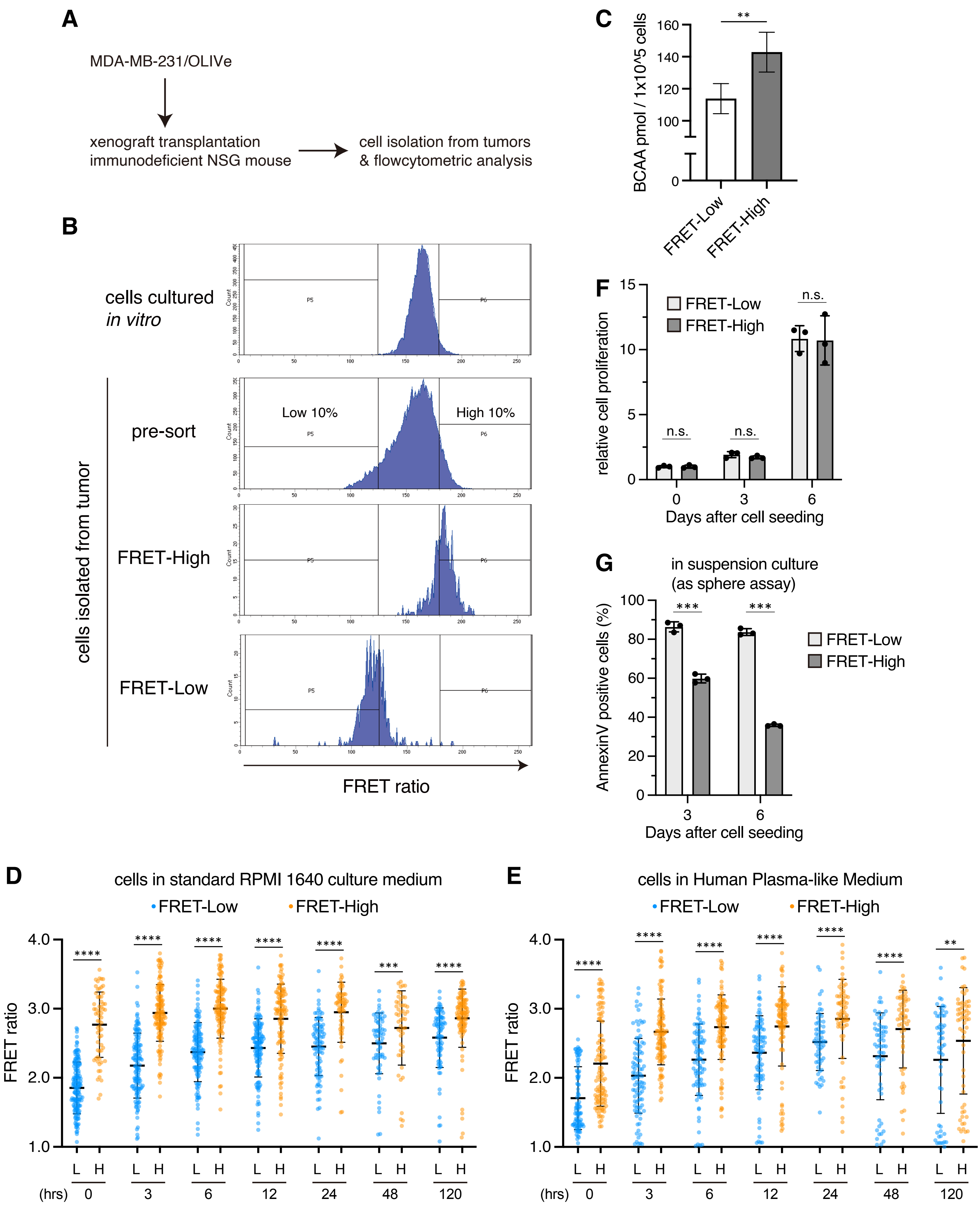

A
MDA-MB-231/OLIVe
xenograft transplantation
immunodeficient NSG mouse
cell isolation from tumors
& flowcytometric analysis
B
cells cultured
in vitro
cells isolated from tumor
pre-sort
FRET-High
FRET-Low
Low 10%
High 10%
FRET ratio
C
BCAA pmol / 1x10^5 cells
**
FRET-Low
FRET-High
F
relative cell proliferation
FRET-Low
FRET-High
n.s.
Days after cell seeding
G
in suspension culture
(as sphere assay)
AnnexinV positive cells (%)
***
FRET-Low
FRET-High
Days after cell seeding
D
cells in standard RPMI 1640 culture medium
FRET-Low
FRET-High
FRET ratio
(hrs)
E
cells in Human Plasma-like Medium
FRET-Low
FRET-High
FRET ratio
(hrs)

Figure S6: Properties of OLIVe FRET-High and -Low cell populations.

(A) Schematic of xenograft tumor cell isolation and sorting based on the OLIVe FRET ratio. Related to Figures 2A and 2B.

(B) Distribution of the OLIVe FRET ratio after cell sorting. Xenograft tumor cells in the top and bottom 10% of the OLIVe FRET ratio were sorted as FRET-High and -Low cells, respectively. Post-sort FRET ratio patterns confirmed successful separation of the two populations. Results for *in vitro* cultured MDA-MB-231 cells and xenograft tumor cells before sorting (pre-sort) were also shown. Related to Figures 2A and 2B.

(C) Intracellular BCAA levels in OLIVe FRET-High and -Low cells. Tumor cells were directly sorted into 80% methanol to extract cellular metabolites. Samples were processed for amino acid measurement. n = 3 per condition. Data are presented as mean ± SD. Paired two-tailed *t*-test was performed, and the *P* value was 0.0081. Related to Figures 2A and 2B.

(D, E) Stability of the OLIVe FRET ratio after cell sorting. OLIVe FRET-High and -Low cells were fractionated and seeded in (D) standard RPMI 1640 culture medium or (E) Human Plasma-like Medium. OLIVe FRET signals were monitored by time-lapse imaging at 30 min intervals for 120 hrs. Image data at 0, 3, 6, 12, 24, 48, and 120 hrs were analyzed to calculate the FRET ratio. More than 50 cells were analyzed at each time point. “H” and “L” indicate OLIVe FRET-High and -Low, respectively. Data are presented as mean ± SD. Unpaired two-tailed Mann-Whitney test was performed. Related to Figure 2F.

(F, G) Cell proliferation and survival of OLIVe FRET-High and -Low cells. FRET-High and -Low cells were fractionated and plated in RPMI 1640 growth media. After 3 and 6 days of culture, cells were subjected to (F) the CellTiter-Glo2.0 assay and (G) the Annexin V staining assay, as described in the METHOD DETAILS. n = 3 per condition. Data are presented as mean ± SD. Unpaired two-tailed *t*-test with Welch's correction was performed. Related to Figure 2C.

Statistical significance was indicated by asterisks based on *P* values: ** <0.01, *** <0.005, and **** <0.001. n.s., not significant.

Figure S7

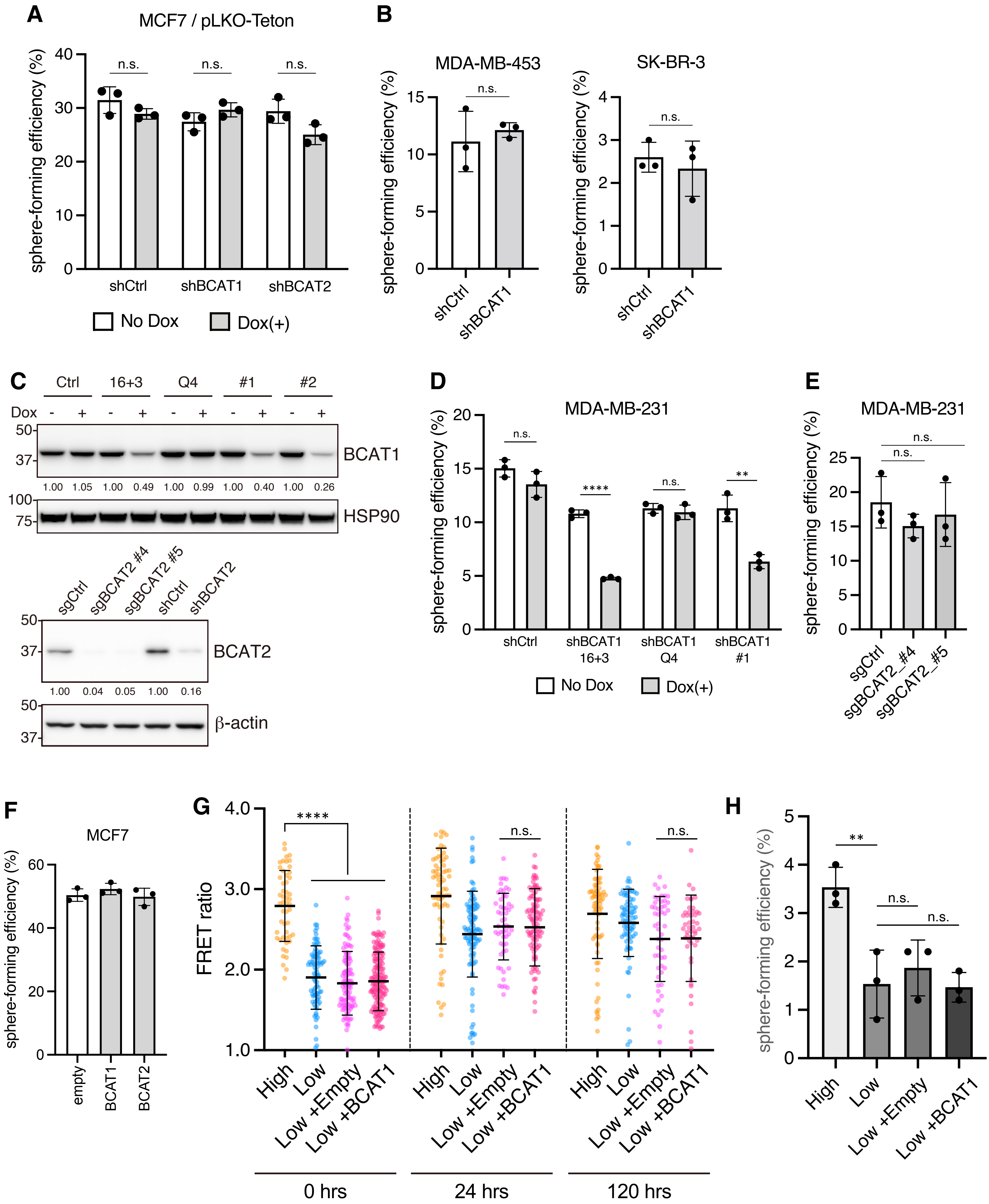
A
MCF7 / pLKO-Teton
sphere-forming efficiency (%)
n.s.
shCtrl
shBCAT1
shBCAT2
No Dox
Dox(+)
B
MDA-MB-453
SK-BR-3
C
Ctrl
16+3
Q4
#1
#2
Dox
BCAT1
1.00 1.05 1.00 0.49 1.00 0.99 1.00 0.40 1.00 0.26
HSP90
sgCtrl
sgBCAT2 #4
sgBCAT2 #5
shCtrl
shBCAT2
BCAT2
1.00 0.04 0.05 1.00 0.16
β-actin
D
MDA-MB-231
shBCAT1 16+3
shBCAT1 Q4
shBCAT1 #1
E
sgBCAT2_#4
sgBCAT2_#5
F
MCF7
empty
BCAT1
BCAT2
G
FRET ratio
High
Low
Low +Empty
Low +BCAT1
0 hrs
24 hrs
120 hrs
H

Figure S7: BCAT1/2 contribution to clonogenic growth and BCAA production in breast cancer cells.
(A) Sphere-forming efficiency with BCAT suppression in MCF7 cells. n = 3 per condition. Data are presented as mean ± SD. One-way ANOVA with Tukey's multiple-comparison test was performed. Related to Figure 3F.
(B) Sphere-forming efficiency with BCAT1 suppression in HER2-positive MDA-MB-453 and SK-BR-3 cells. n = 3 per condition. Data are presented as mean ± SD. Unpaired two-tailed *t*-test with Welch's correction was performed. Related to Figures 3E, and 3F.
(C) Immunoblot analysis of BCAT1 and 2 after knockout/knockdown using independent sh- and sgRNA constructs. Results for four Tet-inducible shBCAT1 constructs, two sgBCAT2 CRISPR constructs, and one shBCAT2 construct are shown. Numbers under each lane indicate relative protein levels. shBCAT1_#2 is the construct primarily used in most knockdown experiments in this study unless otherwise noted. Related to Figures 3E, and 3F.
(D, E) Sphere-forming efficiency of MDA-MB-231 cells with genetic loss of (D) BCAT1 or (E) BCAT2, using the constructs described in (C). n = 3 per condition. Data are presented as mean ± SD. One-way ANOVA with Tukey's multiple-comparison test was performed. Related to Figures 3E, and 3F.
(F) Sphere-forming efficiency with BCAT overexpression in MCF7 cells. MCF7 cells were transduced with lentiviral vectors to overexpress BCAT1 or BCAT2 and seeded for sphere formation assays. n = 3 per condition. Data are presented as mean ± SD. Related to Figures 3G, and 3H.
(G, H) BCAT1 overexpression in OLIVe FRET-Low cells. (G) OLIVe FRET signals were acquired by time-lapse imaging for 120 hrs. Image data of >50 cells at 0, 24, and 120 hrs were analyzed to calculate the FRET ratio. Data are presented as mean ± SD. Welch's ANOVA with Dunnett's T3 multiple-comparison test was performed. (H) Sphere-forming efficiency with BCAT overexpression. 500 cells were seeded, and spheres were counted after 7 days of culture. n = 3 per condition. Data are presented as mean ± SD. One-way ANOVA with Tukey's multiple-comparison test was performed..
Statistical significance was indicated by asterisks based on *P* values: ** <0.01, and **** <0.001. n.s., not significant.

Figure S8

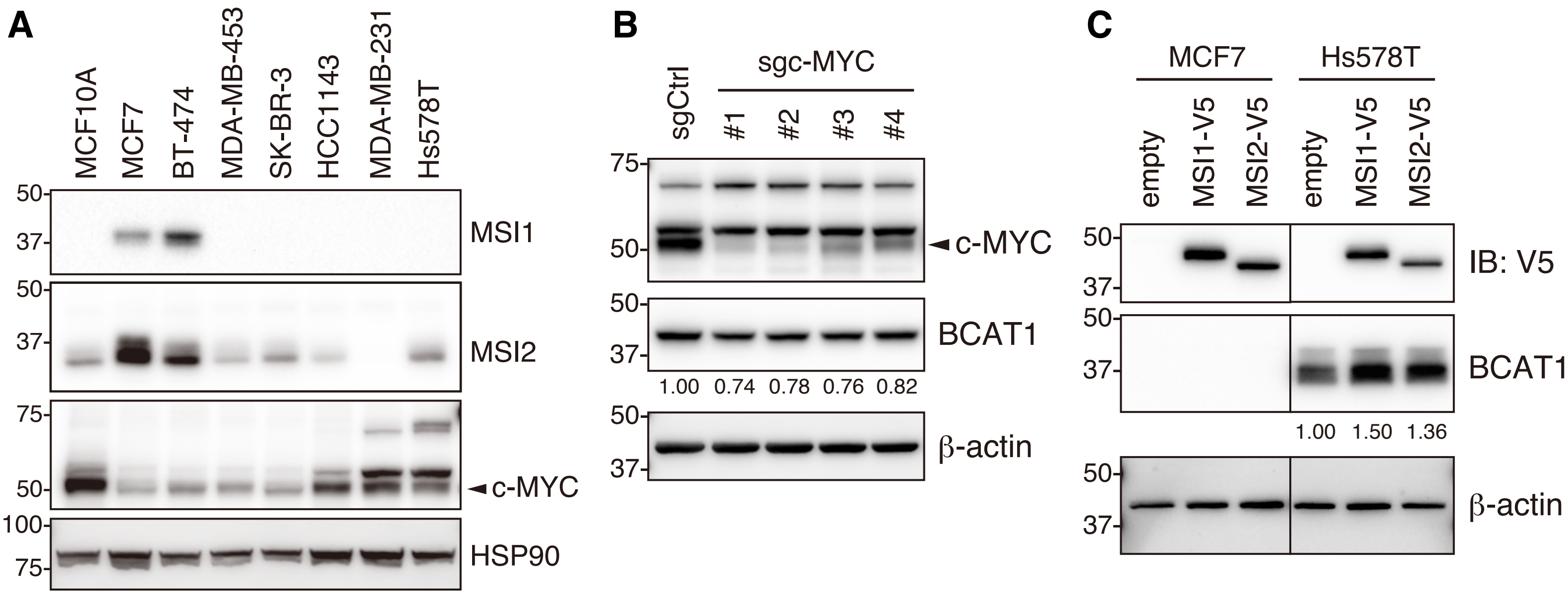

Figure S8: Roles of MSI1/2 and c-MYC in BCAT1 regulation.

(A) MSI1/2 and c-MYC expression in breast cancer cell lines. Protein expression of the RNA-binding proteins MSI1 and MSI2 and the transcription factor c-MYC was assessed by immunoblot. Related to Figure 3B.

(B) c-MYC suppression of BCAT1 expression in CL-TNBC cells. c-MYC was knocked out using four independent target sequences in MDA-MB-231 cells, and c-MYC and BCAT1 protein levels were examined by immunoblot. β-actin served as a loading control. Relative BCAT1 expression levels are shown under each lane. Related to Figure 3B.

(C) MSI1/2 overexpression upregulates BCAT1 expression in CL-TNBC cells. Plasmids encoding V5-tagged MSI1 or MSI2 were transiently transfected into MCF7 and Hs578T cells. After 3 days of culture, BCAT1 expression was assessed by immunoblot. β-actin served as a loading control. Relative BCAT1 expression levels are shown under each lane. Related to Figure 3B.

Figure S9

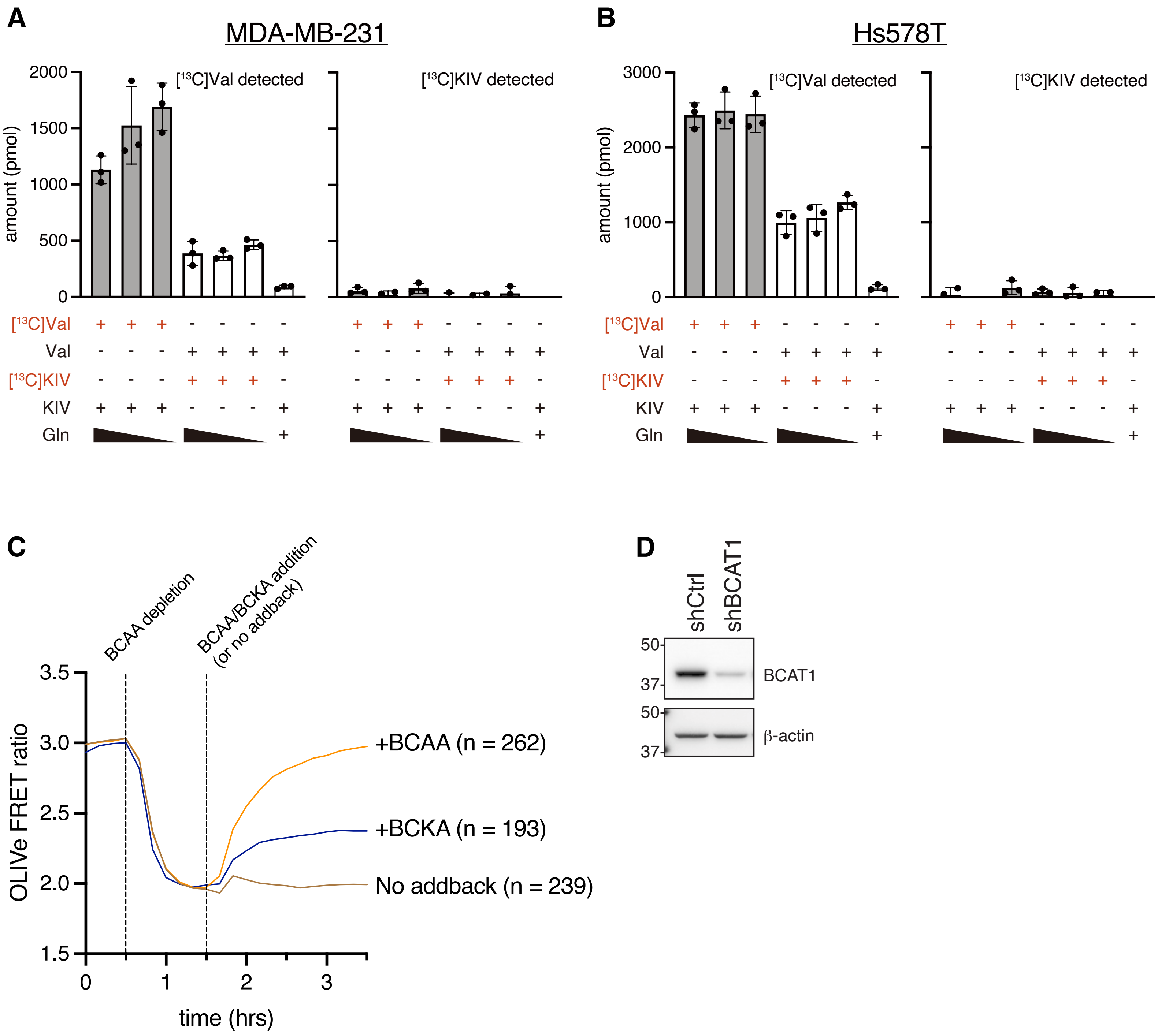

Figure S9: Intracellular conversion of BCKA to BCAA in claudin-low TNBC cells.

(A, B) MDA-MB-231 and Hs578T were cultured with ^{13}C-labeled valine and unlabeled KIV, or with ^{13}C-labeled KIV and unlabeled valine for 30 min. Metabolites were extracted with 80% methanol, vacuum-dried, and analyzed for ^{13}C-incorporated valine or KIV measurements using ^{1}H-^{13}C HSQC NMR. n = 3 for each condition. Data are presented as mean ± SD. Related to Figure 4B.

(C) Time course of the OLIVe FRET signals following the addition of BCAAs or BCKAs in OLIVe-expressing MDA-MB-231 cells. Cells were cultured in the absence of BCAAs for 1h, then either BCAAs or BCKAs were added. Fluorescent signals were acquired at 10-min intervals for 3.5 hrs to calculate FRET ratios as described in Figure S5D. n = 239 for No addback, n = 262 and 239 for BCAA and BCKA addback conditions, respectively. Mean values are shown. Related to Figure 4C.

(D) BCAT1 knockdown efficiency at the protein level. Related to Figure 4D.

Figure S10

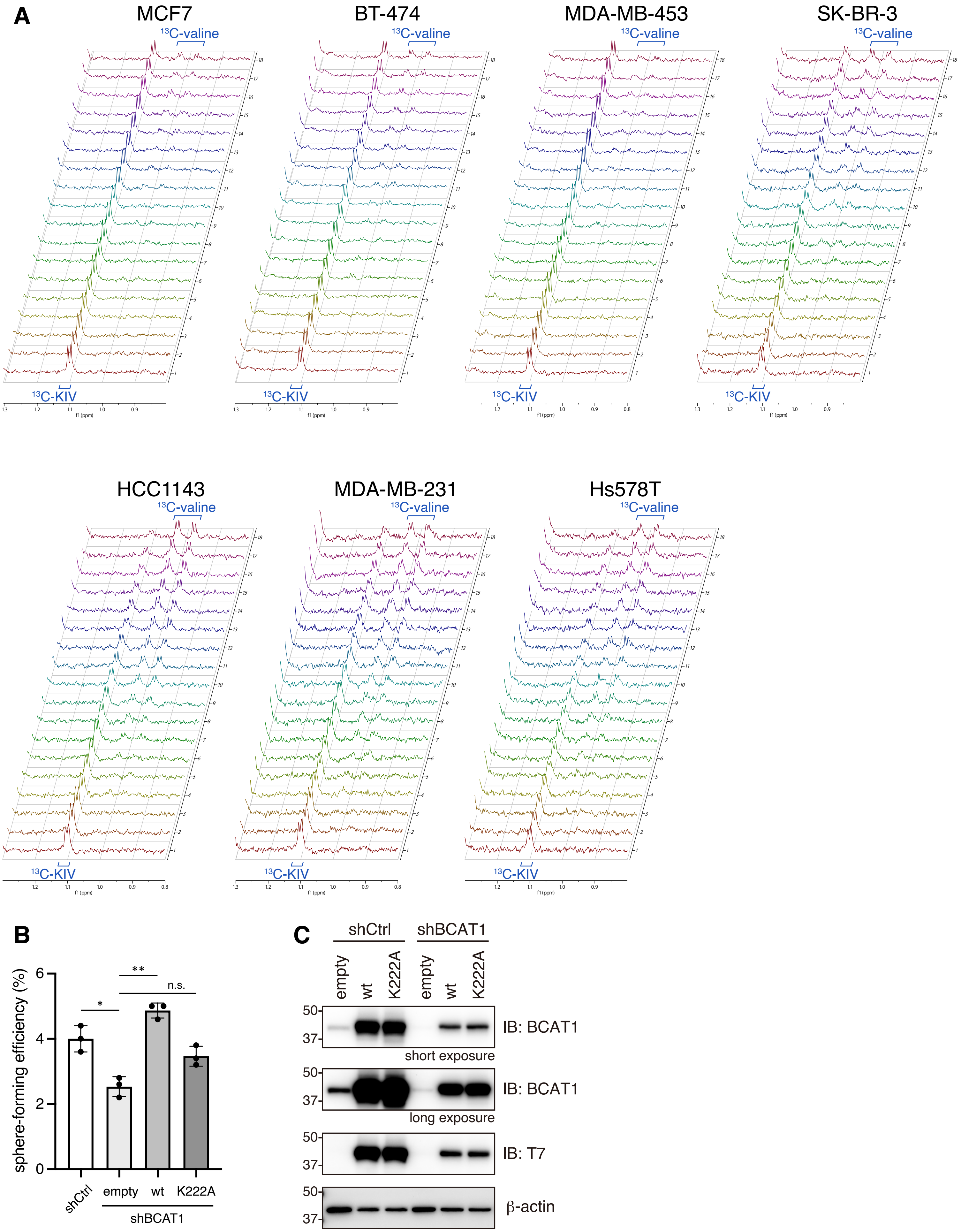
A
MCF7
BT-474
MDA-MB-453
SK-BR-3
HCC1143
MDA-MB-231
Hs578T
13C-valine
13C-KIV
f1 (ppm)
B
sphere-forming efficiency (%)
*
**
n.s.
shCtrl
empty
wt
K222A
shBCAT1
C
shCtrl
shBCAT1
empty
wt
K222A
IB: BCAT1
short exposure
IB: BCAT1
long exposure
IB: T7
β-actin
50
37

Figure S10: Real-time monitoring of and functional dependence on BCKA transamination in breast cancer cells.
(A) Visualizing BCKA-to-BCAA conversion in live breast cancer cells in real time by operando NMR spectroscopy. Right before initiating spectral acquisition, ^{13}C-KIV, at a final concentration of 170 μM, was added to the cell suspension of the indicated breast cancer cells in an HR-MAS rotor. Spectra were acquired over 18 cycles, with each cycle recorded every 2 min (64 scans per cycle). Marked are the chemical shift regions corresponding to the methyl group doublets of ^{13}C-KIV (at 1.11 ppm) and ^{13}C-valine (at 0.98 and 1.03 ppm). Related to Figure 4B.
(B, C) BCAT1 transaminase activity is required for sphere-forming ability in CL-TNBC. (B) T7-epitope-tagged wild-type BCAT1 (wt), transamination-defective mutant (K222A), or a vector alone (empty) was overexpressed in MDA-MB-231 cells with Dox-inducible BCAT1 knockdown, and sphere formation assays were conducted. n = 3 per condition. Data are presented as mean ± SD. One-way ANOVA with Tukey's multiple-comparison test was performed. (C) BCAT1 expression was assessed by immunoblot. Related to Figure 4E.
Statistical significance was indicated by asterisks based on *P* values: * <0.05, and ** <0.01. n.s., not significant.

Figure S11

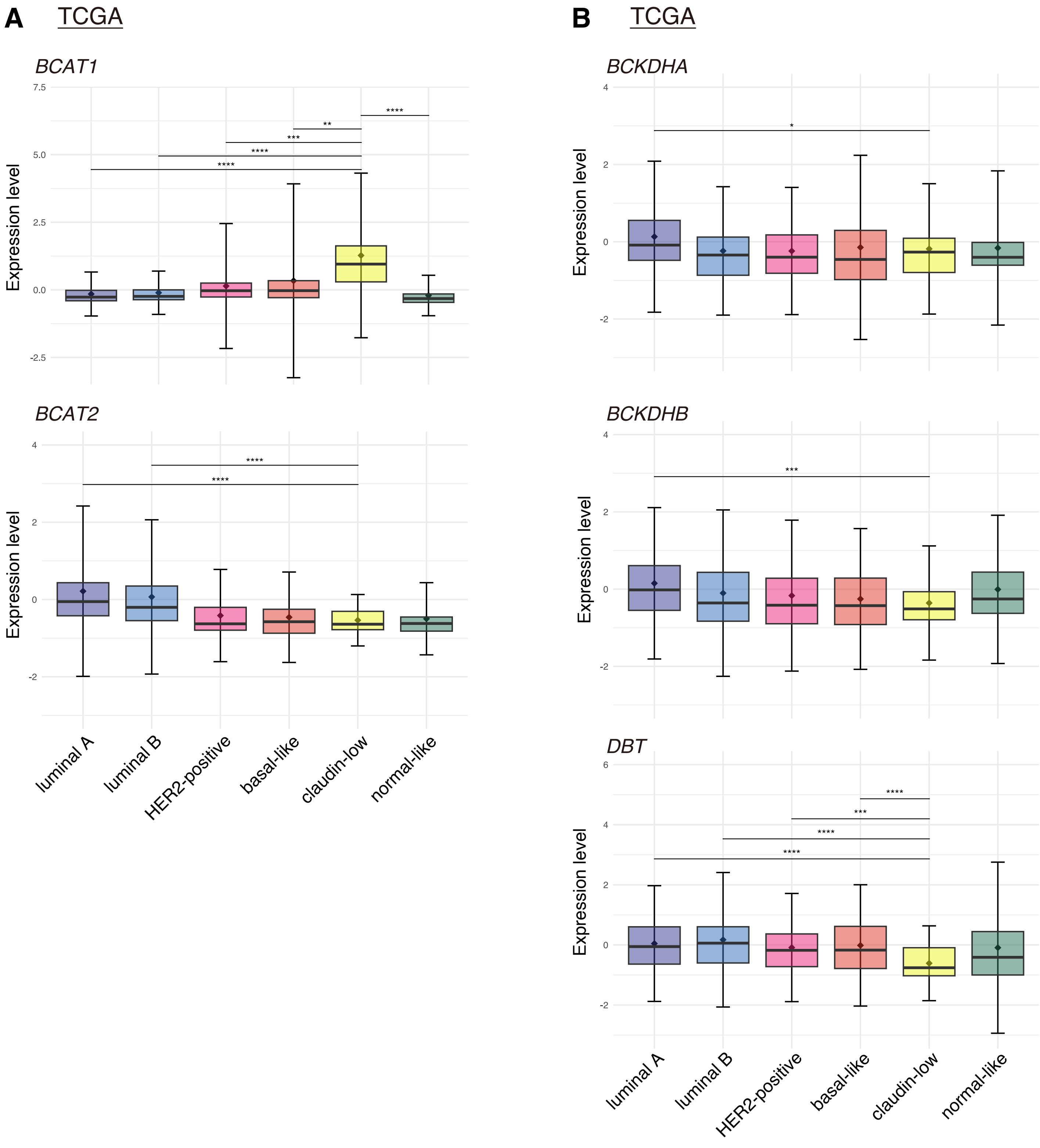

Figure S11: Gene expression analysis of BCAA metabolic pathway components in breast cancer patients using the TCGA dataset.

(A) BCAT1/2 expression. Related to Figure 5A. (B) Expression of genes encoding BCKDH subunits. Related to Figure 6D. Results are organized by breast cancer subtypes as indicated: luminal A (n = 491), luminal B (n = 195), HER2-positive (n = 77), basal-like (n = 162), claudin-low (n = 32), and normal-like (n = 28). Data are shown as box plots with the median (center line) and mean (black diamond). Whiskers represent mean ± 2 × SD. Welch's *t*-test was used for pairwise comparisons between claudin-low and each of the other subtypes.

Statistical significance was indicated by asterisks based on *P* values: * <0.05, ** <0.01, *** <0.005, and **** <0.001.

Figure S12

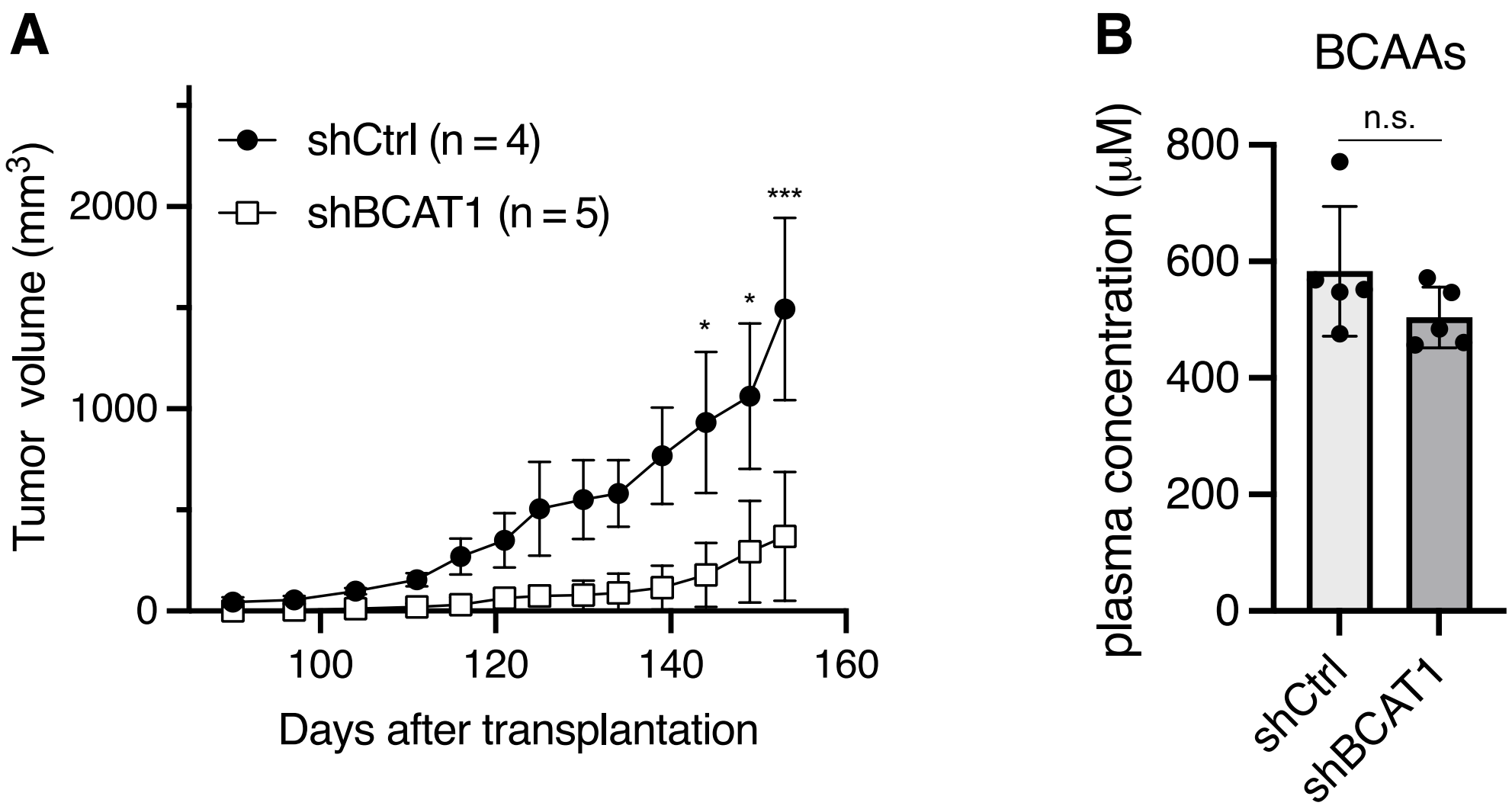

Figure S12: BCAT1 function in tumor growth *in vivo*.

(A) A biological replicate of xenograft tumor growth with BCAT1 knockdown. The experimental design was the same as in Figure 5B. n = 4 and n = 5 for shCtrl and shBCAT1, respectively. Tumor volumes are shown as mean value with SEM. Two-way ANOVA with Sidak's multiple-comparison test was performed. Related to Figures 5B, 5C, and 5D.

(B) Plasma BCAA concentration in tumor-bearing mice with BCAT1 knockdown. Peripheral blood samples were collected from the shCtrl or shBCAT1 groups at 12 weeks post-transplantation and processed for amino acid quantification. n = 5 per group. Data are presented as mean ± SD. Unpaired two-tailed *t*-test was performed. Related to Figures 5B, 5C, and 5D.

Statistical significance was indicated by asterisks based on *P* values: * <0.05, and *** <0.005. n.s., not significant.

Figure S13

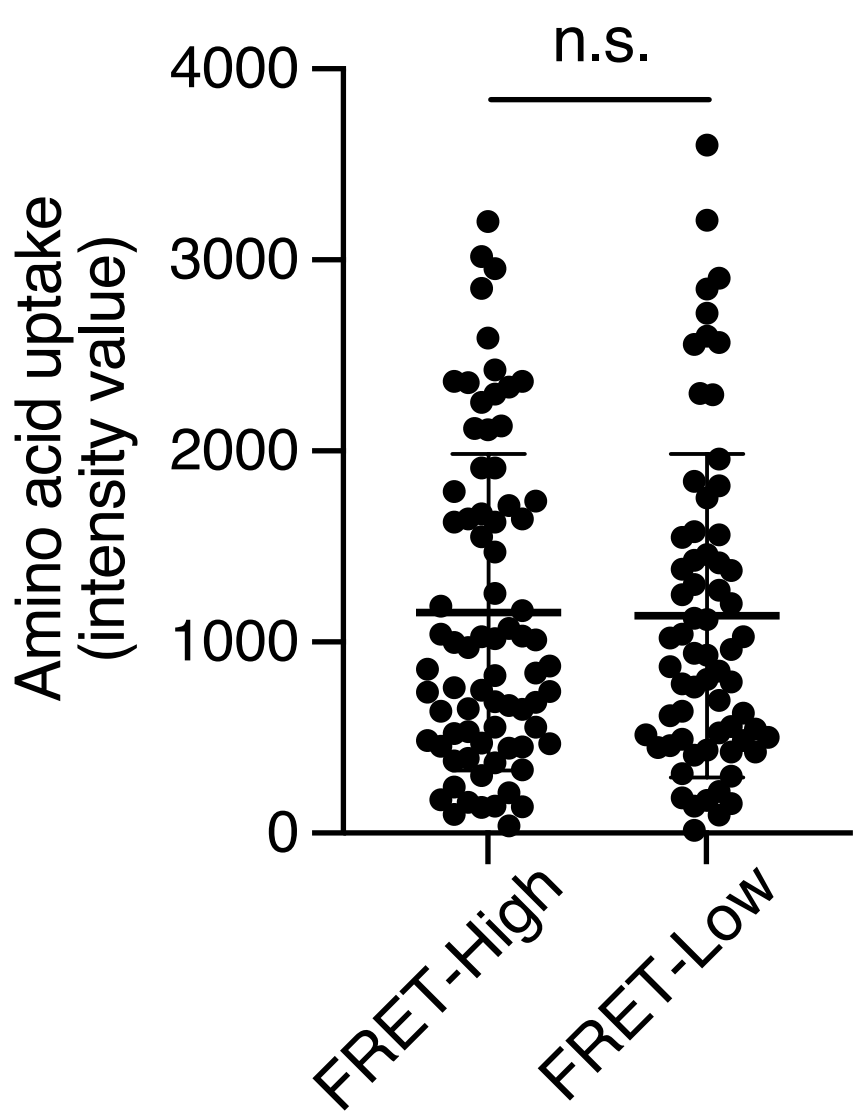

Figure S13: BCAA uptake in OLIVe FRET-High and -Low cells.
OLIVe FRET-High and -Low cells were sorted and plated in cell culture dishes with the amino acid derivative boronophenylalanine (BPA) for 5 min, then detected as described in the METHOD DETAILS section. Fluorescence from the probe-BPA complex was captured and quantitatively analyzed using Fiji software. n = 76 and n = 66 for the FRET-High and -Low groups, respectively. Data are presented as mean ± SD. Unpaired two-tailed *t*-test with Welch's correction was performed.
n.s., not statistically significant.

Figure S14

A Gene Set Enrichment Analysis

Group	Gene_set_name	Count	GeneRatio	pvalue	p.adjust	qvalue
Up	E2F targets	73	0.297	1.91E-43	8.77E-42	8.03E-42
Up	G2M checkpoint	66	0.268	7.34E-36	1.69E-34	1.55E-34
Up	Interferon alpha response	27	0.110	9.29E-13	1.42E-11	1.30E-11
Up	Mitotic spindle	32	0.130	3.24E-08	3.37E-07	3.09E-07
Up	Interferon gamma response	32	0.130	3.66E-08	3.37E-07	3.09E-07
Down	Hypoxia	82	0.398	2.11E-61	9.71E-60	7.11E-60
Down	TNFa signaling via NFkB	49	0.238	1.31E-23	3.01E-22	2.20E-22
Down	Glycolysis	38	0.184	2.35E-14	3.60E-13	2.64E-13
Down	mTORC1 signaling	28	0.136	1.13E-07	1.30E-06	9.53E-07
Down	Apoptosis	19	0.092	0.0002	0.0015	0.0011
Down	Epithelial mesenchymal transition	21	0.102	0.0004	0.0025	0.0018
Down	KRas signaling up	21	0.102	0.0004	0.0025	0.0018
Down	Estrogen response early	20	0.097	0.0010	0.0048	0.0035
Down	p53 pathway	20	0.097	0.0010	0.0048	0.0035
Down	UV response up	17	0.083	0.0010	0.0048	0.0035
Down	IL2 STAT5 signaling	19	0.092	0.0022	0.0094	0.0069
Down	IL6 JAK STAT3 signaling	10	0.049	0.0071	0.0254	0.0186
Down	UV response dn	14	0.068	0.0072	0.0254	0.0186

B

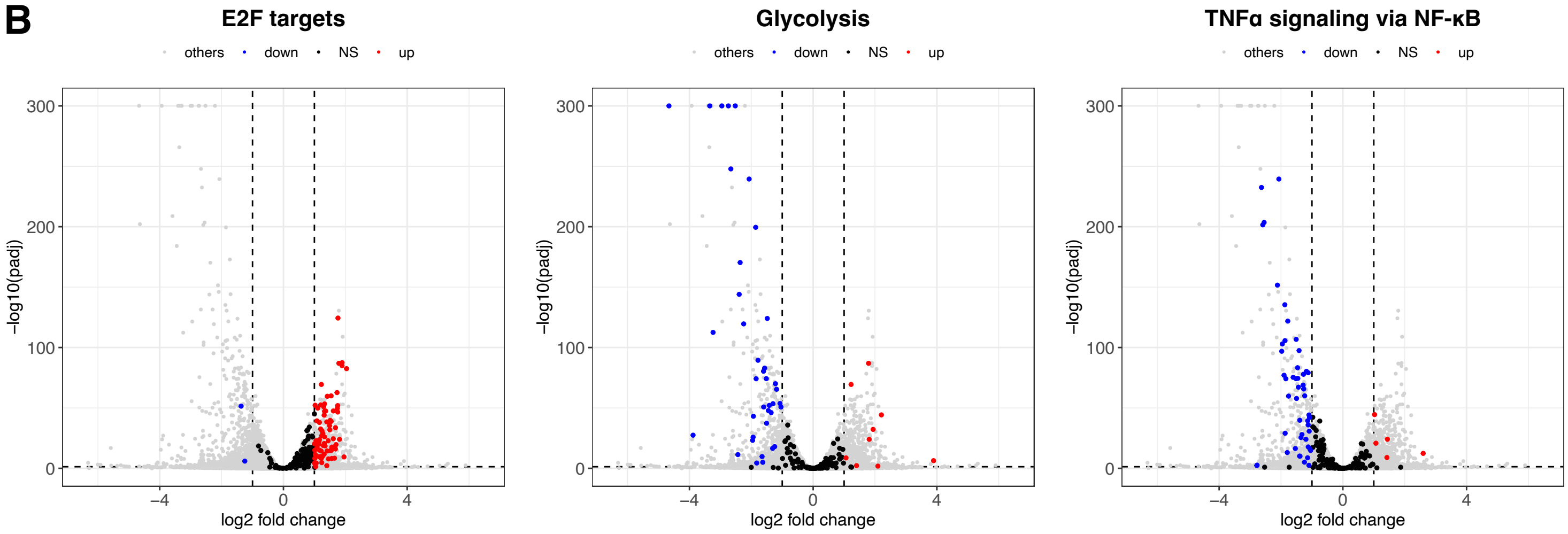

Figure S14: Transcriptomic analysis of OLIVe FRET-High and -Low populations.
(A, B) OLIVe FRET-High and -Low cells were fractionated and subjected to RNA-seq to identify differentially expressed genes.
(A) Gene Set Enrichment Analysis using MSigDB identifies gene sets that are up- or down-regulated in the FRET-High population.
(B) Genes in the indicated gene set are highlighted in the volcano plot. Upregulated genes in the FRET-High and -Low populations are shown as red and blue dots, respectively. Related to Figure 6C.

Figure S15

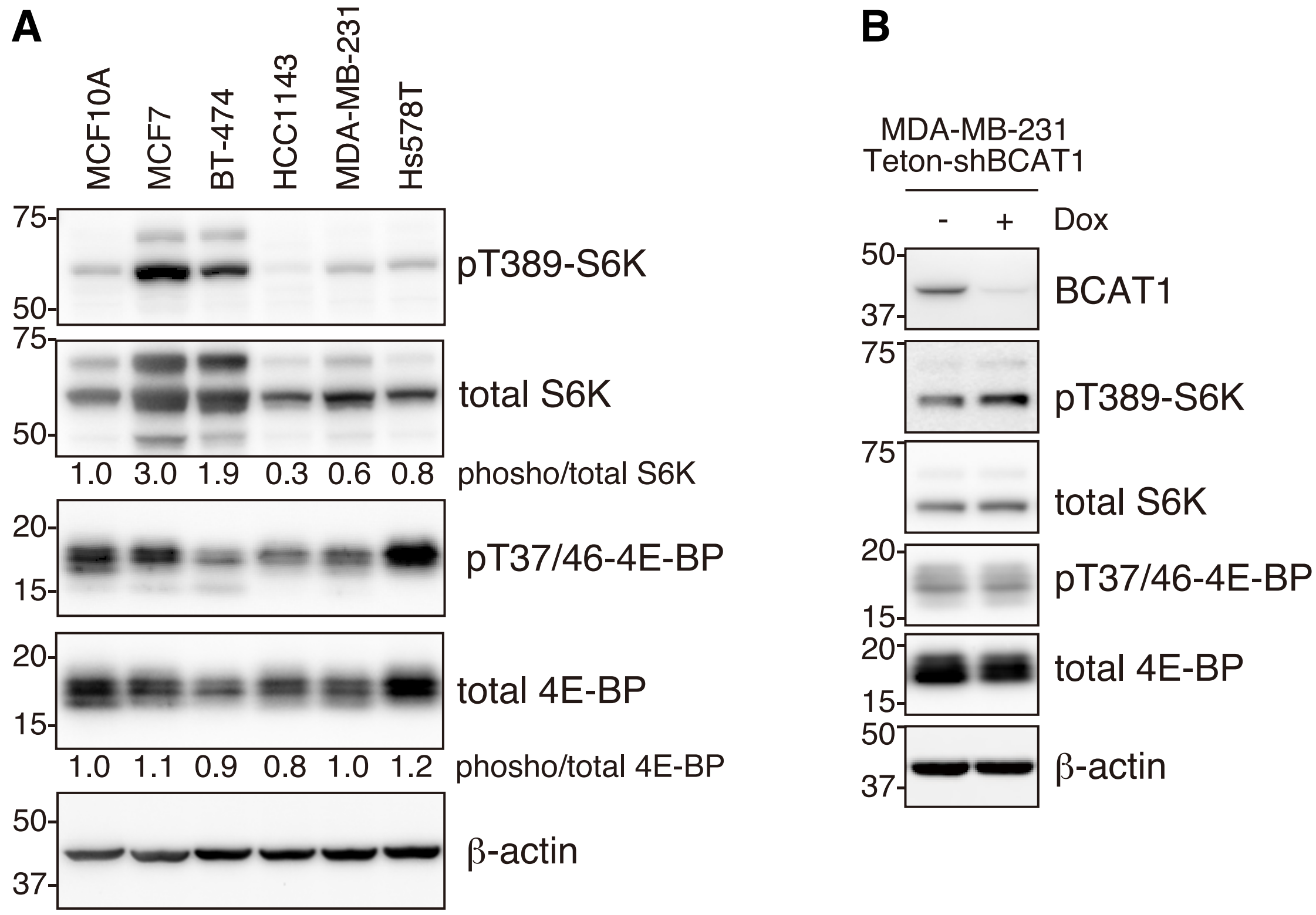

Figure S15: mTORC1 activity in breast cancer cells.

(A, B). Phosphorylation status of S6K and 4E-BP proteins was assessed by immunoblotting as molecular indicators of mTORC1 activity in (A) human breast cancer cell lines and (B) MDA-MB-231 cells with BCAT1 knockdown. β-actin served as a loading control. Fractions of the phosphorylated forms are calculated and shown under each lane. Related to Figure 6B.

Figure S16

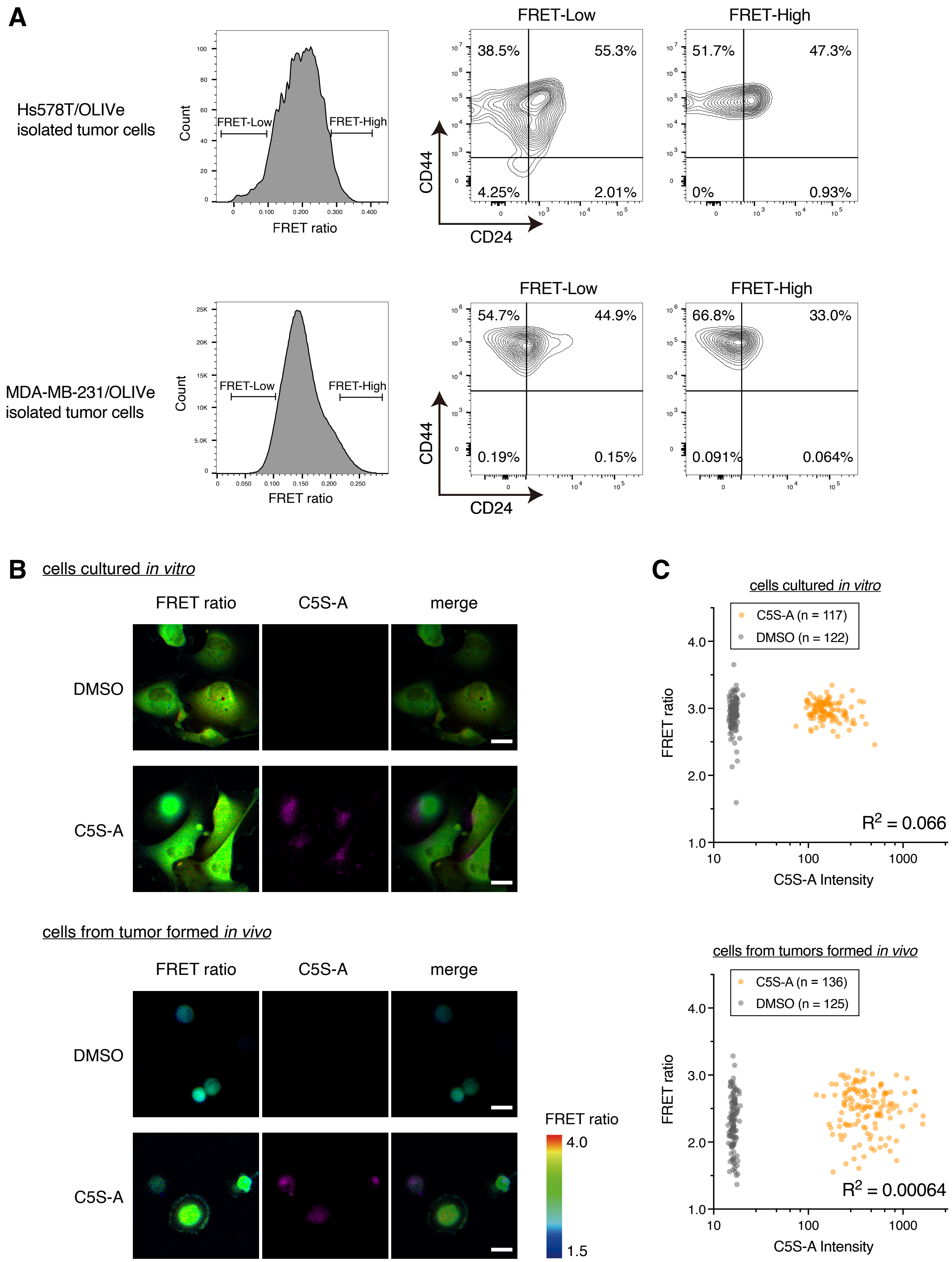

A
Hs578T/OLIVe isolated tumor cells
Count
FRET-Low
FRET-High
FRET ratio
FRET-Low
FRET-High
38.5%
55.3%
4.25%
2.01%
51.7%
47.3%
0%
0.93%
CD44
CD24
MDA-MB-231/OLIVe isolated tumor cells
Count
FRET-Low
FRET-High
FRET ratio
FRET-Low
FRET-High
54.7%
44.9%
0.19%
0.15%
66.8%
33.0%
0.091%
0.064%
CD44
CD24
B
cells cultured in vitro
FRET ratio
C5S-A
merge
DMSO
C5S-A
cells from tumor formed in vivo
FRET ratio
C5S-A
merge
DMSO
C5S-A
FRET ratio
4.0
1.5
C
cells cultured in vitro
C5S-A (n = 117)
DMSO (n = 122)
FRET ratio
R2 = 0.066
C5S-A Intensity
cells from tumors formed in vivo
C5S-A (n = 136)
DMSO (n = 125)
FRET ratio
R2 = 0.00064
C5S-A Intensity

Figure S16: Relationships between intracellular BCAA levels and canonical stem cell markers.
(A) Cells isolated from xenograft tumors generated using OLIVe-expressing Hs578T or MDA-MB-231 were analyzed by flow cytometry for CD44 and CD24 expression. The top and bottom 5% of the FRET ratio population were gated as OLIVe FRET-High and -Low, respectively.
(B, C) OLIVe-expressing MDA-MB-231 cells, cultured *in vitro* or isolated from xenograft tumors, were stained with the ALDH activity probe C5S-A, and fluorescence was acquired on a Zeiss AxioObserver7 fluorescence microscope. DMSO served as the vehicle control. (B) Representative images. Scale bars indicate 20 μm. (C) FRET ratios and C5S-A signal intensities were analyzed and quantified using the Fiji application. Dot plots show the correlation between the FRET ratio and C5S-A fluorescence for each cell. Pearson's correlation coefficients (R^2) are shown. The number of cells analyzed in each group is shown in the inset.
